# Phylogeny- and Abundance-Based Metrics Allow for the Consistent Comparison of Core Gut Microbiome Diversity Indices Across Host Species

**DOI:** 10.3389/fmicb.2021.659918

**Published:** 2021-05-11

**Authors:** Alice Risely, Mark A. F. Gillingham, Arnaud Béchet, Stefan Brändel, Alexander C. Heni, Marco Heurich, Sebastian Menke, Marta B. Manser, Marco Tschapka, Wasimuddin, Simone Sommer

**Affiliations:** ^1^Institute of Evolutionary Ecology and Conservation Genomics, University of Ulm, Ulm, Germany; ^2^Institut de Recherche de la Tour du Valat, Le Sambuc, Arles, France; ^3^Smithsonian Tropical Research Institute, Ancon, Panama; ^4^Department of Visitor Management and National Park Monitoring, Bavarian Forest National Park, Grafenau, Germany; ^5^Chair of Wildlife Ecology and Management, University of Freiburg, Freiburg, Germany; ^6^Institute for Forest and Wildlife Management, Inland Norway University of Applied Sciences, Koppang, Norway; ^7^Department of Evolutionary Biology and Environmental Studies, University of Zurich, Zurich, Switzerland

**Keywords:** host-microbe communities, gut microbiota, core microbiome, community ecology, methods, bioinformatics

## Abstract

The filtering of gut microbial datasets to retain high prevalence taxa is often performed to identify a common core gut microbiome that may be important for host biological functions. However, prevalence thresholds used to identify a common core are highly variable, and it remains unclear how they affect diversity estimates and whether insights stemming from core microbiomes are comparable across studies. We hypothesized that if macroecological patterns in gut microbiome prevalence and abundance are similar across host species, then we would expect that increasing prevalence thresholds would yield similar changes to alpha diversity and beta dissimilarity scores across host species datasets. We analyzed eight gut microbiome datasets based on 16S rRNA gene amplicon sequencing and collected from different host species to (1) compare macroecological patterns across datasets, including amplicon sequence variant (ASV) detection rate with sequencing depth and sample size, occupancy-abundance curves, and rank-abundance curves; (2) test whether increasing prevalence thresholds generate universal or host-species specific effects on alpha and beta diversity scores; and (3) test whether diversity scores from prevalence-filtered core communities correlate with unfiltered data. We found that gut microbiomes collected from diverse hosts demonstrated similar ASV detection rates with sequencing depth, yet required different sample sizes to sufficiently capture rare ASVs across the host population. This suggests that sample size rather than sequencing depth tends to limit the ability of studies to detect rare ASVs across the host population. Despite differences in the distribution and detection of rare ASVs, microbiomes exhibited similar occupancy-abundance and rank-abundance curves. Consequently, increasing prevalence thresholds generated remarkably similar trends in standardized alpha diversity and beta dissimilarity across species datasets until high thresholds above 70%. At this point, diversity scores tended to become unpredictable for some diversity measures. Moreover, high prevalence thresholds tended to generate diversity scores that correlated poorly with the original unfiltered data. Overall, we recommend that high prevalence thresholds over 70% are avoided, and promote the use of diversity measures that account for phylogeny and abundance (Balance-weighted phylogenetic diversity and Weighted Unifrac for alpha and beta diversity, respectively), because we show that these measures are insensitive to prevalence filtering and therefore allow for the consistent comparison of core gut microbiomes across studies without the need for prevalence filtering.

## Introduction

Host-associated gut microbial communities often comprise thousands of taxa, most of which are rare, and therefore are challenging and computationally intensive to analyze (Pollock et al., 2018; Hornung et al., 2019; Pascoal et al., 2021). One solution for simplifying analyses and to potentially clarify biological patterns is to focus on a “common core” gut microbiome, which is broadly defined as the suite of common gut microbes that are shared across host individuals that are assumed to have biological relevance to the host (Turnbaugh et al., 2009; Shade and Handelsman, 2012; Risely, 2020). The core microbiome is a concept founded in community ecology to describe the common bimodal pattern in macrobes where many species fall into the highest and lowest occupancy classes, and can be easily distinguished as either “core” or “satellite” species (Hanski, 1982). This bimodal pattern is generally not found in microbial communities, however, making the distinction between core and satellite species more challenging, if not impossible. As such, common core membership is often inferred by arbitrary thresholds in taxa prevalence (Shade and Handelsman, 2012; Risely, 2020), defined as the proportion of host individuals in which the taxa was detected (sometimes referred to as “occupancy frequency” or “occurrence” in macroecology), independent of its abundance within hosts. This approach stems from the assumption that host-adapted microbes coevolve with the host in a manner that promotes their colonization and persistence in most individuals (Round et al., 2011; Lee et al., 2013; Shapira, 2016; Shukla et al., 2018). In contrast, taxa with low prevalence may represent transient microbes that shift with environmental variables or early yet stochastic colonizers, and make up the non-core component of gut microbial communities (Martínez et al., 2013; Shapira, 2016; Obadia et al., 2017).

Identifying a common core via prevalence filtering is a common methodological approach that has frequently been used to investigate the connection between gut microbiota composition, and host biology and co-evolution (Ainsworth et al., 2015; e.g., Cheng et al., 2016; Falony et al., 2016; Jeffery et al., 2016; Stephens et al., 2016; Grieneisen et al., 2017; Sevellec et al., 2018; Amato et al., 2019; Gibson et al., 2019; Wallace et al., 2019). Filtering microbiome data to identify a common core differs conceptually from quality filtering (“denoising”) and statistical filtering based on statistical reliability, which aim to exclude sequencing errors and taxa that cannot be reliably analyzed, respectively. Quality filtering by excluding very low prevalence taxa (e.g., that occur in just a few samples) reduces effects of sequencing error (Bokulich et al., 2013; Callahan et al., 2016; Amir et al., 2017), although it is argued this method also excludes rare yet real taxa and therefore can bias results in other ways (Kozich et al., 2013; Jousset et al., 2017; Schloss, 2020). Statistical filtering, on the other hand, removes rare yet resident taxa (i.e., they are not due to sequencing error) and is recommended for many analyses, such as network and differential abundance analysis to increase their reliability (Röttjers and Faust, 2018; Cougoul et al., 2019; Cao et al., 2021). Statistical filters generally apply higher prevalence thresholds than quality filters, with taxa below ∼20% prevalence often being limited in their statistical testability, although this number is dependent on sample size (Cougoul et al., 2019). It should be noted that extremely high prevalence also limits testability, and that applying abundance data instead of prevalence data adds little value to these testability thresholds (Cougoul et al., 2019). In contrast, filtering for a common core aims to retain only prevalent taxa that are assumed to have biological relevance to the host (Shade and Handelsman, 2012), and usually involves higher prevalence thresholds than is required for statistical reliability, often between 30 and 90% (Ainsworth et al., 2015; Grieneisen et al., 2017; Mahnic and Rupnik, 2018; Gibson et al., 2019). Whilst the appropriate thresholds for quality and statistical filtering are often testable with, e.g., power analyses, identifying a common core often applies more arbitrary thresholds that may be dependent on the aims to the study and dataset characteristics (Shade and Handelsman, 2012; although see Shade and Stopnisek, 2019).

Because prevalence thresholds for identifying core gut microbiomes are often based on untestable assumptions of taxa biological function to the host, prevalence thresholds applied across studies are highly variable, and it remains unclear whether results are comparable. If microbial communities demonstrate predictable macroecological properties in prevalence and abundance distributions (e.g., Grilli, 2020; Ji et al., 2020), it is reasonable to assume that increasing prevalence thresholds may have comparable effects on alpha and beta diversity metrics across microbial datasets. If so, studies that utilize the common core concept are likely to be comparable, and guidelines that improve cross-study comparison and interpretation are possible. Currently, such guidelines are hindered by our limited understanding of the extent to which alpha diversity and beta dissimilarity decrease with increasing prevalence values, and whether such affects are consistent across datasets and diversity indices. Understanding these patterns has implications for the choice of prevalence threshold and community metrics used in community-level analyses across different host species, as well as cross-study standardization. However, prevalence estimates require numerous host individuals sampled per population, and gut microbial datasets consisting of such sample sizes for wild populations are relatively rare, therefore this question has not been formally tested across datasets.

To examine the effect of increasing prevalence thresholds on alpha diversity and beta dissimilarity across gut microbial datasets, we analyzed 16S rRNA gene amplicon-based microbiome data of 1,970 individuals from humans and seven species of wild mammals and birds. These datasets vary in their sampling protocol and host species ecology, yet are characterized by reasonably high frequency sampling of individuals (between 98 and 552), therefore allowing us to estimate reasonably precise prevalence and abundance distributions. Our study has three aims: (1) to compare macroecological patterns of gut microbiomes collected from diverse host species, including examining differences in amplicon sequence variant (ASV) detection rate with sequencing depth and sample size, and differences in occupancy-abundance and rank-abundance curves; (2) to test whether increasing prevalence thresholds to identify a core microbiome generates universal or host-species specific effects on eight measures of alpha and beta diversity that vary in how they weight ASV abundance and phylogeny (Table 1); and (3) to test whether diversity scores from filtered core communities correlate with the original unfiltered data. Together, these results facilitate the analysis of common core gut microbiomes by providing baseline information on ASV distributions and the downstream effects of prevalence filtering across a broad range of host species.

**TABLE 1:** Definitions and descriptions for alpha and beta diversity measures applied in this study.

Diversity measure	Index	Weighting	Description	References
**Alpha**	Observed richness	Not weighted	Number of ASVs detected per sample	NA
	Faith’s PD	Phylogeny-weighted	Sum of the branch lengths of the phylogenetic tree connecting all microbial taxa present within a sample	Daniel, 1992; Chao et al., 2015
	Shannon	Abundance-weighted	A diversity index based on the number of ASVs present and their abundance distribution (evenness)	Shannon, 1948
	BWPD	Phylogeny- and abundance-weighted	Abundance-weighted extension of phylogenetic diversity	McCoy and Matsen, 2013
**Beta**	Jaccard	Not weighted	Variability in microbial composition among sampled communities, with composition measured by which ASVs are present or absent	Jaccard, 1912
	Unweighted Unifrac	Phylogeny-weighted	Variability in microbial composition among sampled communities based on the lineages they contain	Lozupone and Knight, 2005
	Morisita	Abundance-weighted	Variability in microbial composition among sampled communities based on ASV presence and abundance. Sensitive to the most abundant species	Morisita, 1959; Chao et al., 2006
	Weighted Unifrac	Phylogeny- and abundance-weighted	Abundance-weighted extension of Unweighted Unifrac	Lozupone et al., 2007

## Materials and Methods

### Datasets

Gut microbiota 16S rRNA gene amplicon data from humans and seven wildlife species were analyzed from raw sequence data and metadata per species dataset are outlined in Table 2. These datasets include publicly available human gut microbiome samples from the American Gut Project study (*n* = 500) and data collected by authors as part of various ecological studies, such as meerkats *Suricata suricatta* from South Africa (*n* = 137), red deer from Germany *Cervus elaphus* (*n* = 136), Seba’s short-tailed bat *Carollia perspicillata* from Panama (*n* = 169), Tome’s spiny rat *Proechimys semispinosus* from Panama (*n* = 196), gray-brown mouse lemur *Microcebus grisorufus* from Madagascar (*n* = 182), greater flamingo *Phoenicopterus roseus* juveniles (2–4 months old) from three breeding sites in France and Spain (*n* = 552), and red-necked stint *Calidris ruficollis* from Australia (*n* = 98). Samples collected from meerkats, red deer, *Carollia* bats, and spiny rats were collected from sites within small geographic areas spanning approximately 20 km or under. Flamingo juveniles were sampled at three separate breeding areas in southern France and Spain. Stint were sampled over a large geographic range on the northern-western and southern coasts of Australia. Temporally, red deer samples were collected within 1 week, but meerkats, *Carollia* bats, spiny rats, mouse lemurs and stint samples were collected over approximately 2 years or more. Details on each dataset, including site of collection, sample type and preservation, 16S rRNA gene hypervariable regions amplified, read counts, associated publications, and data storage on public repositories from where sequences can be downloaded are outlined in Table 2.

**TABLE 2:** Metadata associated with each dataset.

Species	Latin name	Country	No. of samples	Sample type	Sample buffer	16S Primers	Mean read count per sample	Associated publication	Data availability
Humans	*Homo sapiens*	United States	500	Feces	None	515F/806R (V4)	33,454	American Gut Project	NCBI BioProject PRJEB11419
Meerkat	*Suricata suricatta*	South Africa	137	Feces	None/RNAlater	515F/806R (V4)	129,011	NA	NCBI BioProject PRJNA715730
Red deer	*Cervus elaphus*	Germany	136	Feces	RNAlater	515F/806R (V4)	48,667	Menke et al., 2019	https://doi.org/10.5061/dryad.7r22vb1
Seba’s short-tailed bat	*Carollia perspicillata*	Panama	169	Feces	RNAlater	515F/806R (V4)	36,549	NA	NCBI BioProject PRJNA715730
Tome’s spiny rat	*Proechimys semispinosus*	Panama	196	Feces	RNAlater	515F/806R (V4)	25,045	Fackelmann et al., 2021	NCBI BioProject PRJNA715350
Gray-brown mouse lemur	*Microcebus grisorufus*	Madagascar	182	Feces	RNAlater	515F/806R (V4)	49,910	Wasimuddin et al., 2019	NCBI BioProject PRJNA715730
Greater flamingo	*Phoenicopterus roseus*	France	552	Cloacal swab	RNAlater	515RF/806R (V4)	27,970	Gillingham et al., 2019	NCBI BioProject PRJNA485732
Red-necked stint	*Calidris ruficollis*	Australia	98	Cloacal swab	None	27F/519R (V1-3)	42,573	Risely et al., 2018	NCBI BioProject PRJNA385545

### DNA Extraction, PCR Amplification, and 16S rRNA Sequencing

DNA was extracted from fecal samples for all species except for flamingos and red-necked stint, for which cloacal swabs were used. Detailed DNA extraction and PCR protocols for humans, flamingos, red deer, mouse lemur, and red-necked stint can be found in their associated publications (McDonald et al., 2018; Risely et al., 2018; Gillingham et al., 2019; Menke et al., 2019; Wasimuddin et al., 2019). Samples from meerkats, red deer, spiny rats, mouse lemur, and flamingos were processed at University of Ulm using the following protocol: DNA was extracted from fecal samples using either the Qiagen Cador Pathogen extraction kit (Qiagen, Hilden, Germany; flamingo samples), or the NucleoSpin Soil Kit (Macherey-Nagel, Germany; samples from all other species) following the manufacturer’s instructions. This protocol includes a bead-beating step to mechanically lyse bacterial cells using ceramic beads that was carried out using the SpeedMill PLUS (Analytik Jena, Germany) following manufacturer’s instructions. Polymerase chain reaction (PCR) amplification and barcoding were conducted in two steps (two-step PCR). In the first step, the 291 bp fragment of the hypervariable V4 region located in the 16S rRNA gene was targeted using the universal bacterial primers 515F (5’-GTGCCAGCMGCCGCGGTAA-3’) and 806R (5’-GGACTACHVGGGTWTCTAAT-3’), appended with forward-primer CS1 adapters (CS1-515F) and reverse-primer CS2 adapters (CS2-806R) in order to use Fluidigm chemistry (Access Array System for Illumina Sequencing Systems, Fluidigm Corporation). PCR reactions of 10 μL consisted of 200 nM primers (pooled forward and reverse primers), 5 μL AmpliTaq Gold 360 Master Mix, 1 μL extracted DNA sample, 1 μL DNA template (5–10 ng), and dH_2_0. PCR conditions were as follows: initial denaturation at 95°C for 10 min, 30 cycles at 95°C for 30 s for denaturation, 60°C for 30 s for annealing, and 72°C for 45 s for elongation, followed by a final elongation at 72°C for 10 min. In the second PCR step, the CS adapters were attached to sample-specific primer pairs that contained 10 bp barcodes and adapter sequences used for Illumina sequencing. PCR reactions of 20 μL consisted of 4 μL (400 nM) barcode primers (pooled forward and reverse primers), 10 μL AmpliTaq Gold 360 Master Mix, 3 μL amplified DNA from PCR step one, and dH_2_0. PCR conditions were as above, but included 10 cycles instead of 30. For red-necked stint samples, DNA was extracted from swabs using the phenol-chloroform method (Risely et al., 2018), and the V1-V3 region was amplified with the primer pair 27F (5′-AGAGTTTGATCMTGGCTCAG-3′) and 519R (5′-GWATTACCGCGGCKGCTG-3′; Table 2). DNA was amplified and sequenced at the Ramaciotti Centre for Genomics, Sydney, following Earth Microbiome Project 16S protocol (Thompson et al., 2017). Samples for humans were collected and processes as part of the American Gut Project, and DNA extraction and PCR amplification of the V4 region followed standard EMP protocols (McDonald et al., 2018). For all datasets, amplicons were sequenced with Illumina MiSeq technology over 2 × 250 cycles.

### Bioinformatics

All sequence reads were processed using QIIME2 (Bolyen et al., 2019). For all datasets except for humans, sequences were merged, quality filtered, and chimera filtered using the DADA2 pipeline (Callahan et al., 2016) to generate ASVs (amplicon sequence variants that differ by one nucleotide; Callahan et al., 2017). For the human dataset, merged sequences were downloaded. Human sequences were therefore treated as single end reads and processed with DADA2 for quality and chimera filtering, for consistency with paired-end reads. ASVs were assigned a taxonomy using SILVA (release 132). A tree was built using FastTree 2.1.8 (Price et al., 2010) for phylogenetic analyses. An archaeal sequence (accession number: KU656649) was used to root the tree and was removed prior to analysis. ASVs were filtered if they were not bacteria, not assigned to a phylum (as these are assumed to be spurious), or if they were classified as mitochondria or chloroplasts at the family and class level, respectively. DADA2 automatically discards singletons, but no other ASVs were excluded. Only samples that had over 10,000 reads post filtering were retained and presented here, in order to minimize the effect of low read counts on results.

### Data Analysis

We first aimed to test whether the species datasets demonstrated similar macroecological patterns, including ASV detection rates with sequencing depth and sample size. Measuring ASV detection rates allows us to assess how reliable ASV prevalence and abundance distributions are. To this end, we generated ASV accumulation curves with sequencing depth per sample and accumulation curves with sample size per dataset to assess how well rare (both in terms of prevalence and abundance) ASVs were represented. We used the *vegan:specaccum* function (Jari Oksanen et al., 2018) with 999 permutations and the *ranacapa:ggrare* function to generate accumulation curves on unnormalized data. We next wanted to predict the total number of ASVs harbored by the sampled individuals of a species, in order to estimate the proportion of ASVs that remained undetected. To do this we used the *vegan:specpool* function, applying the “Jackknife 1” method. Because accumulation curves of percent ASVs detected (out of predicted total number of ASVs) seemed to differ markedly between datasets, we investigated this further by testing how sample size affects estimates of the overall ASV pool of a species, using the *vegan:poolaccum* function, and linked this to the proportion of ASVs that only occur in one individual. Lastly, we compared occupancy-abundance curves and rank abundance curves between species datasets.

Our second aim was to examine the effect of increasing prevalence thresholds on within-individual alpha diversity and beta dissimilarity scores. We first rarefied each dataset to 10,000 to control for differences in sequencing depth between datasets, and rarefying is proposed to be an appropriate normalization method for alpha and beta diversity analyses (Weiss et al., 2017; McKnight et al., 2019). We repeated analyses without normalizing, which generated similar results. In addition, core microbiomes are usually identified from rarefied datasets (Grieneisen et al., 2017; Russell et al., 2019), and therefore this method reflects common practice. We next subsetted the rarefied dataset by prevalence at 10% intervals (all ASVs, 10, 20, 90%). We calculated four alpha diversity metrics (Table 1) that accounted for observed diversity (number of ASVs), phylogenetic-weighted diversity (Faith’s phylogenetic diversity), abundance-weighted diversity (Shannon index), and abundance- and phylogenetic-weighted diversity (balance-weighted phylogenetic diversity (BWPD); McCoy and Matsen, 2013). Alpha diversity scores were mean centered and scaled per species, using the scale function, in order to account for natural differences in alpha diversity between species, because our aim is to understand relative effects of prevalence thresholds on gut microbiomes rather than absolute effects.

We next calculated mean beta-diversity dissimilarity scores for each individual applying four different measures of beta diversity (Table 1; Anderson et al., 2011). As with our choice of alpha diversity indices, we applied a metric that does not account for either abundance or phylogeny (i.e., only presence/absence of taxa, Jaccard), one that just account for abundance (Morisita), one that just accounts for phylogeny (Unweighted Unifrac), and one that accounts for both abundance and phylogeny (Weighted Unifrac). We chose the Morisita index over the more commonly applied Bray-Curtis index because Bray-Curtis was almost perfectly correlated with Jaccard, and Morisita is more sensitive to abundant species (Barwell et al., 2015) and therefore is more appropriate for detecting the effects of abundant taxa on community structure within large, diverse communities. Beta dissimilarity scores vary between zero (highly similar) and one (highly dissimilar). We tested whether increasing prevalence thresholds affected variation in alpha and beta diversity across samples with Bartlett’s test, which tests whether measures across groups have equal variation. We also tested whether the number of reads remaining after prevalence filtering correlated with diversity measures using Spearman’s correlation, because variation in read depth post prevalence filtering may bias weighted diversity scores.

Our third aim was to test whether diversity scores of gut core microbiomes correlated with the original unfiltered datasets. We correlated alpha and beta diversity scores at each prevalence threshold with scores from the original, unfiltered dataset using Spearmans correlation. We extracted Spearman’s rho (effect size) and *p*-values and plotted effect sizes by species dataset.

All diversity analyses were carried out using the packages phyloseq (McMurdie and Holmes, 2013) vegan (Jari Oksanen et al., 2018), metagMisc^1^, btools^2^, picante (Kembel et al., 2010), and microbiome (Lahti et al., 2017). All analyses were carried out in R version 3.5.3. R code and processed data are available to download at https://github.com/Riselya/Prevalence-Thresholds-Metaanalysis, and an R markdown document outlining the analysis is available as Supplementary Data Table 1.

## Results

### ASV Detection Rate and Macroecological Curves

**FIGURE 1:**
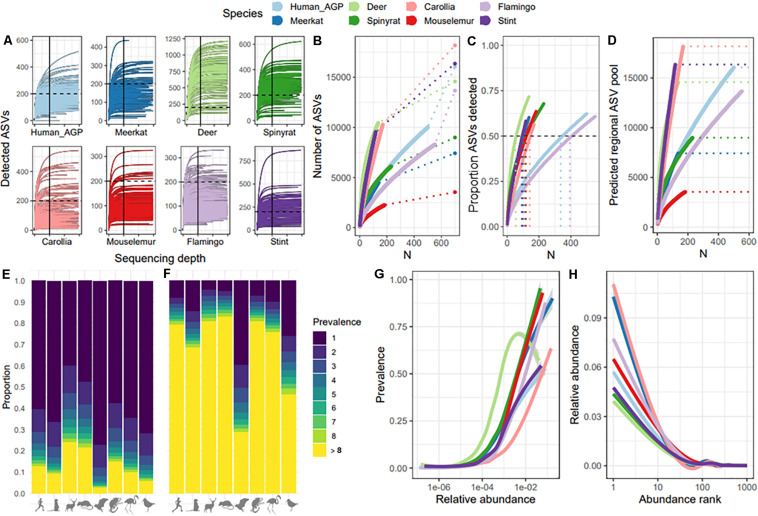
Comparison of ASV detection rates and macroecological patterns across species datasets. **(A)** Rarefaction curves per species dataset, showing ASV detection with increasing sequencing depth per sample. To facilitate comparison, the 200 ASV mark is represented by a dashed line, and 10,000 reads is indicated with a solid line. *X*-axis ticks mark every 10,000. **(B)** ASV accumulation curves with sample size, showing the extent to which each additional sample increases total number of ASVs detected per species dataset. Dashed lines represent extrapolations to the total number of ASVs predicted to be within the overall ASV pool, represented by end points. **(C)** Percent of total (predicted) ASVs detected with increasing sample size per species dataset. The dashed horizonal line marks 50% of ASVs detected, whilst the vertical dashed lines represent the sample size required to detect 50% of predicted ASVs. **(D)** The relationship between sample size and predictions of the overall ASV pool. Dashed lines represent the final ASV pool prediction per dataset, which match those shown in Figure 1A. **(E)** ASV prevalence distribution per dataset, showing the proportion of ASVs found in just one sample (dark blue) to the proportion found in over eight samples (yellow). **(F)** ASV prevalence distribution per sample, showing mean proportion of ASVs per sample found in just that sample (dark blue) to proportion found in at least eight other samples (yellow). **(G)** Abundance-occupancy curves per dataset. **(H)** Rank-abundance curves per species dataset.

We first compared species datasets to test whether sequencing depth per sample and sampling depth per dataset adequately captured within-sample and population-level ASV pools, respectively. For all datasets, ASV accumulation curves showed that ASV detection leveled off at a sequencing depth of 10,000, indicating that sequence depth is not a limiting factor for ASV detection beyond 10,000 reads (Figure 1A). Comparisons of ASV accumulation curves according to sample size revealed a large divergence between datasets, with some showing very shallow curves (e.g., mouse lemurs and spiny rats) and others showing very steep curves with no indication of leveling off (e.g., *Carollia* bats and red-necked stint; Figure 1B). We estimated the total ASV pool for each dataset (dashed lined and final point in Figure 1B) which suggested that approximately 50–60% of ASVs predicted to be present in the sampled population were detected across datasets (Figure 1C). However, these predictions assume a closed system where no new ASVs are introduced, therefore are likely to be an underestimate. Further investigation revealed that estimates of total ASV diversity at the host population level are strongly dependent on sample size (Figure 1D), suggesting that each new sample adds a unique suite of ASVs and affects predictions of total ASV diversity. This bias was most severe for *Carollia* bats and red-necked stint datasets, with sample size having very large effects on predictions. We examined the source of this variation and found that whilst most datasets were characterized by a high proportion of ASVs occurring in just one individual (mean 60% of ASVs; Figure 1E), samples from *Carollia* and red-necked stint tended to be much more individualized that those from other species. On average, 39 and 26% of ASVs per sample were unique to each sample for *Carollia* and red-necked stint, respectively, compared to approximately 8% in other datasets (Figure 1F). Therefore, species datasets diverged in their distribution and detection of rare taxa. Despite this divergence, we found that occupancy-abundance (Figure 1G) and rank-abundance (Figure 1H) curves tended to follow similar patterns across species datasets, with the most abundant ASVs making up between 4 and 10% relative abundance, and being detected in 50–90% of samples.

### Effect of Prevalence Threshold on Alpha Diversity and Beta Dissimilarity Scores

**FIGURE 2:**
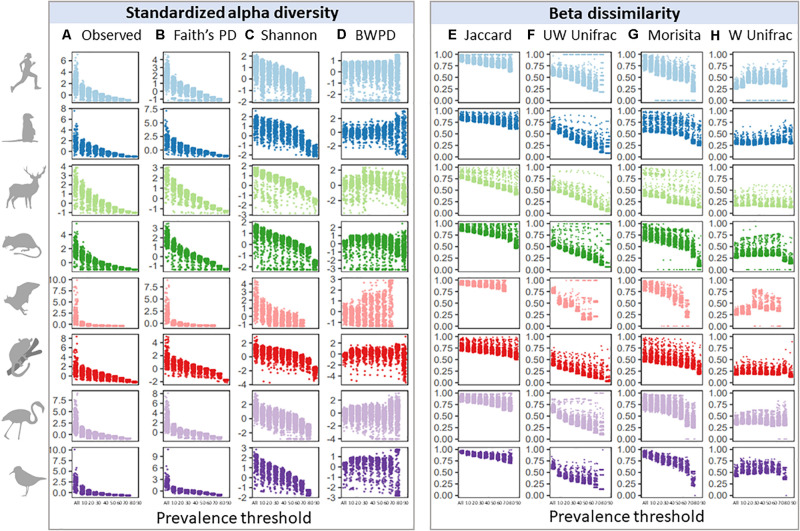
Effects of increasing prevalence threshold on standardized alpha diversity and beta dissimilarity measures, colored by species dataset: **(A)** observed ASV richness; **(B)** faiths phylogenetic diversity; **(C)** shannon index; **(D)** balance-weighted phylogenetic diversity (BWPD); **(E)** jaccard index; **(F)** unweighted Unifrac; **(G)** morisita; **(H)** weighted Unifrac.

Given similar occupancy-abundance and rank-abundance curves across species datasets, we predicted that increasing prevalence thresholds when filtering for a common core would have similar effects on standardized alpha diversity and beta dissimilarity estimates. We first looked at the effect of increasing prevalence thresholds on variation in alpha and beta diversity across samples (Figure 2), since maintaining variation may be a priority for subsequent analyses. Variation across samples decreased with increasing prevalence thresholds for observed and phylogenetic alpha diversity (Figures 2A,B), and to a lesser extent Shannon diversity and BWPD (Figures 2C,D; Barlett’s K-squared: Observed = 23,583; Faiths = 14,616; Shannon = 1,240; BWPD = 2,031, *p* < 2.2e-16). In contrast, variation in beta dissimilarity tended to increase with increasing prevalence thresholds for most measures of beta diversity, with the smallest and largest changes in variance across thresholds observed in Morisita and Unweighted Unifrac, respectively (Figures 2E–H; Barlett’s K-squared: Jaccard = 1,827; Unweighted Unifrac = 2,405; Morisita = 352; Weighted Unifrac = 874, *p* < 2.2e-16). This increase in variance was because a subset of samples became increasingly dissimilar to the mean with increasing prevalence thresholds. To test whether variation in read depth post-prevalence filtering had a consistent bias on abundance-weighted diversity scores, we correlated alpha and beta diversity scores with sample read depth and found no consistent pattern across species datasets (Supplementary Figure 1). Higher read depth per sample after filtering was associated with higher weighted alpha diversity scores in some host species (e.g., mouse lemurs), and lower weighted alpha diversity scores in others (e.g., meerkats), and this was also the case for weighted beta diversity measures (Morisita and Weighted Unifrac). For presence/absence diversity measures, higher read depth was associated with higher alpha diversity scores, but lower beta diversity scores (i.e., higher read samples tended to lie close to the group centroid whilst low-read samples tended to be outliers).

**FIGURE 3:**
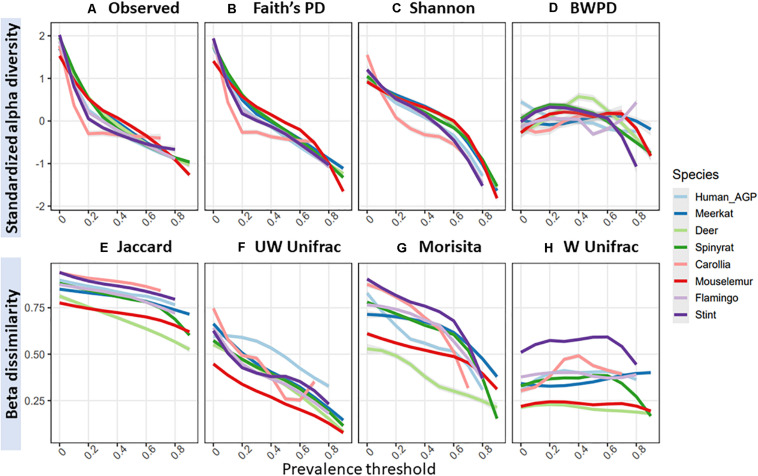
Mean standardized alpha diversity and beta dissimilarity measures with increasing prevalence thresholds, colored by species dataset: **(A)** observed ASV richness; **(B)** faiths phylogenetic diversity; **(C)** shannon index; **(D)** balance-weighted phylogenetic diversity (BWPD); **(E)** jaccard index; **(F)** unweighted Unifrac; **(G)** morisita; **(H)** weighted Unifrac.

Across species datasets, patterns in standardized alpha diversity and beta dissimilarity in response to increasing prevalence thresholds were remarkably similar (Figure 3). Overall, most alpha diversity metrics were highly sensitive to increasing prevalence thresholds, with the exception of BWPD, which was the least sensitive to increasing prevalence thresholds. BWPD responses to prevalence thresholds tended to diverge at approximately 70% (Figure 3D). Species datasets demonstrated variation in their mean beta diversity (i.e., how similar sampled individuals are to each other), but followed similar patterns in their response to increasing prevalence thresholds (Figures 3E–H). Unweighted Unifrac and Morisita values were the most sensitive to increasing prevalence thresholds, with samples becoming more similar to one another (Figures 3F,G), whilst Weighted Unifrac was the least sensitive, with samples remaining relatively similar to one another irrelevant of prevalence threshold (Figure 3D).

**FIGURE 4:**
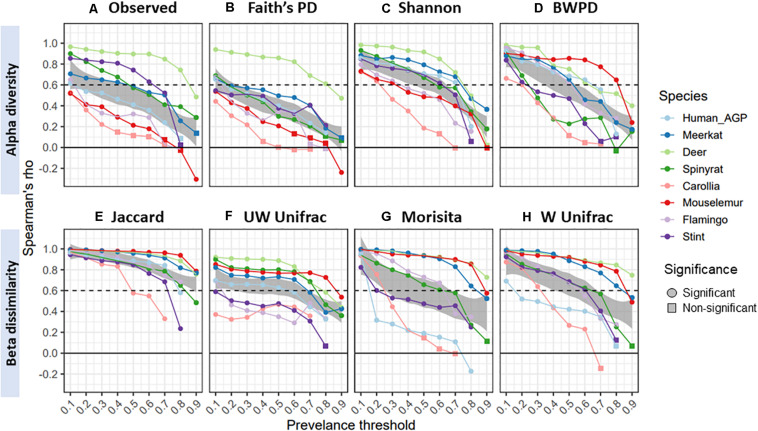
Spearman’s correlation (rho) between diversity scores from core microbiomes and scores from original unfiltered data, colored by species dataset: **(A)** observed ASV richness; **(B)** faiths phylogenetic diversity; **(C)** shannon index; **(D)** balance-weighted phylogenetic diversity (BWPD); **(E)** jaccard index; **(F)** unweighted Unifrac; **(G)** morisita; **(H)** weighted Unifrac. Negative values represent negative correlations, and for ease of interpretation a dashed line represents a correlation of 0.6. Circles represent significant correlations (*p* < 0.05), whilst squares represent non-significant correlations.

Lastly, we examined the extent to which diversity scores from gut core microbiomes correlated with original, unfiltered scores. Spearman’s correlation between core and original diversity scores decreased with prevalence thresholds, declining from an overall mean of 0.83 (±0.16 SD) at 10% prevalence thresholds, to 0.6 (±0.26 SD) and then 0.46 ± 0.29 (SD) at 50 and 70% thresholds, respectively (Figure 4). Rates of decline varied considerably across datasets, with gut microbiomes from red deer maintaining high correlations with original scores, whilst those from *Carollia* having consistently low correlations. Overall, Shannon diversity (mean correlation = 0.58) and Jaccard (mean correlation = 0.85) maintained the highest correlations with the original diversity scores for the alpha and beta diversity measures, respectively.

## Discussion

The gut core microbiome is often identified through arbitrary prevalence thresholds, and we aimed to understand how shifting prevalence thresholds altered alpha and beta diversity scores across gut microbiome datasets sourced from diverse host species We found that increasing prevalence thresholds had highly comparable effects on standardized alpha and beta dissimilarity scores across host species, and that this was underpinned by similar occupancy-abundance and rank-abundance macroecological patterns. Across datasets, the most abundant ASVs made up between 4 and 10% relative abundance, and were detected in 50–90% of samples. However, despite overall similarities in macroecological patterns, numbers of high prevalence taxa varied substantially across species datasets, and therefore alpha diversity and beta dissimilarity tended to become more divergent above 70% prevalence thresholds, or were not even measurable. Moreover, we found that correlations with the original diversity scores were often low when prevalence thresholds were high, indicating that prevalence thresholds alter sample rankings in terms of diversity scores. Together, these results suggest that high prevalence thresholds (e.g., >70%) generate results that are less likely to be comparable across studies.

Our results confirm that across datasets, high prevalence thresholds dramatically reduce observed and phylogenetic diversity within samples, and increase sample similarity in terms of composition. Moreover, we show that prevalence filtering can have additional disadvantages such as altering sample diversity rankings in a manner that may not be consistent across studies. Therefore, whilst filtering rare taxa can reduce technical bias (Cao et al., 2021), heavier filtering parameters may not always generate ecologically meaningful results that are comparable across the literature (although see Ainsworth et al., 2015; Grieneisen et al., 2017; Russell et al., 2019), and variation in sequencing depth after filtering may also bias weighted alpha and beta diversity scores unless further normalization methods are applied (e.g., Silverman et al., 2017; Beule and Karlovsky, 2020). Our results indicate that diversity measures that account for both abundance and phylogeny (BWPD and Weighted Unifrac, for alpha and beta diversity, respectively) are insensitive to prevalence thresholds, and therefore represent the common core microbiome without the need for filtering. As such, we recommend that where possible these metrics are applied in lieu of prevalence filtering. Alternatively, a core microbiome could be identified by using occupancy-abundance curves (Shade and Stopnisek, 2019), or by using temporal persistence or ecological interactions as a basis for determining taxa importance (Björk et al., 2017; Toju et al., 2018; Risely, 2020).

Applying prevalence thresholds assumes that measures of ASV prevalence distributions are relatively accurate and that rare ASVs are detectable. We tested limitations of ASV detection rates, and found that ASV detection was more likely to be limited by sample size than sequencing depth. For all datasets included here, rarefaction curves with sequencing depth leveled off rapidly, with sequencing depths of over 10,000 reads per sample (after mitochondria and chloroplast filtering) in most cases not improving ASV detection rates, which is line with other studies of 16S rRNA gut data (Zaheer et al., 2018; Gweon et al., 2019). Nevertheless, it should be noted that rare taxa will continue to be discovered at lower detection rates with increasing sequencing depth, and may even then remain undetected to due technical reasons such as extraction and primer bias (Brooks et al., 2015). In contrast, we found that ASV accumulation rates with sample size differed substantially between species datasets, with ASV detection rates with sample size remaining high in most cases (i.e., many ASVs were still being detected with every sample added, even at the point when the final sample was added). Sampled populations characterized by highly individualized microbiomes (i.e., a high proportion of ASVs that only occurred in one sample; in this study *Carollia* bats and migratory red-necked stint), demonstrated particularly steep accumulation curves that did not level off, suggesting further sampling is required to more fully capture the population-level ASV pool of these populations. The extent to which these differences in ASV detection rates reflect sampling protocol or host ecology is unclear, yet individualized microbiomes in some bird and bat species may be underpinned by a lack of strong evolutionary symbioses with gut microbes in these lineages (Song et al., 2020), and therefore may require higher sampling effort to measure population-level ASV diversity.

Whilst ASV accumulation rates with sample size differed among species datasets, our predictions of the total ASV pool hosted by the host population suggested that all datasets detected approximately 50–60% of ASV diversity. The interpretation of this figure is subject to debate, since predicting species diversity in highly diverse microbial communities is extremely challenging, and generally underestimated (Hong et al., 2006). Since we found that predictions for the total ASV pool hosted by the wider population are highly sensitive to sample size (Figure 1D), and that predictions assume a closed system (i.e., no new ASVs are introduced to the microbial community), we interpret this figure as the number of ASVs likely to be harbored in the gut microbiomes of the sampled animals, and also likely represent the majority of non-unique ASVs harbored by the wider host population. Under the assumption that each host harbors at least some unique strains in their gut microbiome as a result of stochastic colonization (Obadia et al., 2017), the total ASV pool of the host population—including strains unique to individuals—is impossible to estimate with any degree of certainty. For example, our knowledge of human gut microbiome diversity is continually increasing: previous research on the human gut microbiome using shotgun sequencing has identified over 60,000 prokaryotic genomes (bacterial and archaeal) from 3,810 samples sourced from people across the globe (Nayfach et al., 2019), whilst another comprehensive global study identified almost 160,000 unique genomes from 9,428 samples (Pasolli et al., 2019), indicating that increasing sampling frequency is still revealing considerable increases in global human gut diversity. For comparison, our analysis of a subsample of 500 samples downloaded from the American Gut Project dataset detected over 10,000 ASVs, with on average 10% of ASVs detected in each sample occurring uniquely. Therefore, our results suggest that whilst increasing sample sizes will increase the accuracy of ASV prevalence distributions, even very large sample sizes can often not sufficiently represent the large suite of rare, low prevalence taxa harbored by the host population, and that the proportion of taxa that only occur in one sample can differ markedly between host populations.

## Conclusion

Our results show that macroecological patterns in occupancy-abundance and rank-abundance curves, and their downstream effects on standardized alpha diversity and beta dissimilarity, are similar across gut microbiome datasets, and therefore studies that apply similar thresholds are likely to be comparable assuming sufficient sampling frequency. However, trends in alpha and beta diversity scores tended to diverge above 70% prevalence thresholds, and diversity scores at high prevalence thresholds tended to correlate poorly with original data. Therefore, setting high prevalence thresholds when filtering microbiome datasets may hinder cross-study comparisons. To reduce downstream effects of prevalence filtering, we recommend the use of diversity metrics that account for both phylogeny and abundance (such as BWPD and Weighted UniFrac), which we show represent the common core microbiome without the need for filtering.

## Data Availability Statement

Sequence data is available at the project numbers and DOIs outlined in Table 2. R code and processed data to replicate study are available to download at https://github.com/Riselya/Prevalence-Thresholds-Metaanalysis. An R markdown document is available as Supplementary Data Sheet 1.

## Ethics Statement

Ethical review and approval were not required for the study on human participants in accordance with the local legislation and institutional requirements. Data are publicly available. All data from other species were collected under the appropriate permits, details of which can be found in the associated publications (Table 2). For data reported for the first time here: Meerkat samples were collected with the permission of the ethical committee of Pretoria University and the Northern Cape Conservation Service, South Africa (Permit number: EC031-13). Spiny rat samples were collected with full ethical approval (Smithsonian Institutional Animal Care and Use Committee protocols 2013-0401-2016-A1-A7 and 2016-0627-2019-A2). Carollia samples were ethically approved by the Smithsonian Tropical Research Institute 144 (IACUC protocols: 2014-0101-2016 and 2016-0627-2019).

## Author Contributions

AR led on conceptualization, performed analyses, and wrote manuscript. MG supported on conceptualization and manuscript editing. SS led on supervision, data management, and supported manuscript editing. AB, SB, MG, AH, MH, SM, MM, MT, and WM contributed to data acquisition and editing manuscript. All authors contributed to the article and approved the submitted version.

## Conflict of Interest

The authors declare that the research was conducted in the absence of any commercial or financial relationships that could be construed as a potential conflict of interest.

## Footnotes

1 https://github.com/vmikk/metagMisc

2 https://github.com/twbattaglia/btools
