# Supplementary material for: Phylogeny- and Abundance-Based Metrics Allow for the Consistent Comparison of Core Gut Microbiome Diversity Indices Across Host Species
Source: Front Microbiol. 2021 May 11;12:659918. doi: 10.3389/fmicb.2021.659918 (PMC8144293; doi:10.3389/fmicb.2021.659918)

Supplementary figure

Phylogeny- and abundance-based metrics allow for the consistent comparison of core gut microbiome diversity indices across host species

Figure S1) The effect of read depth (post filtering) on diversity scores per species and per diversity measure. The figure shows the correlation coefficient (Spearman's rho) between sample read depth and sample diversity, coloured by species and shaped by whether the correlation was significant. The grey smoothed line shows average values when species are considered together.

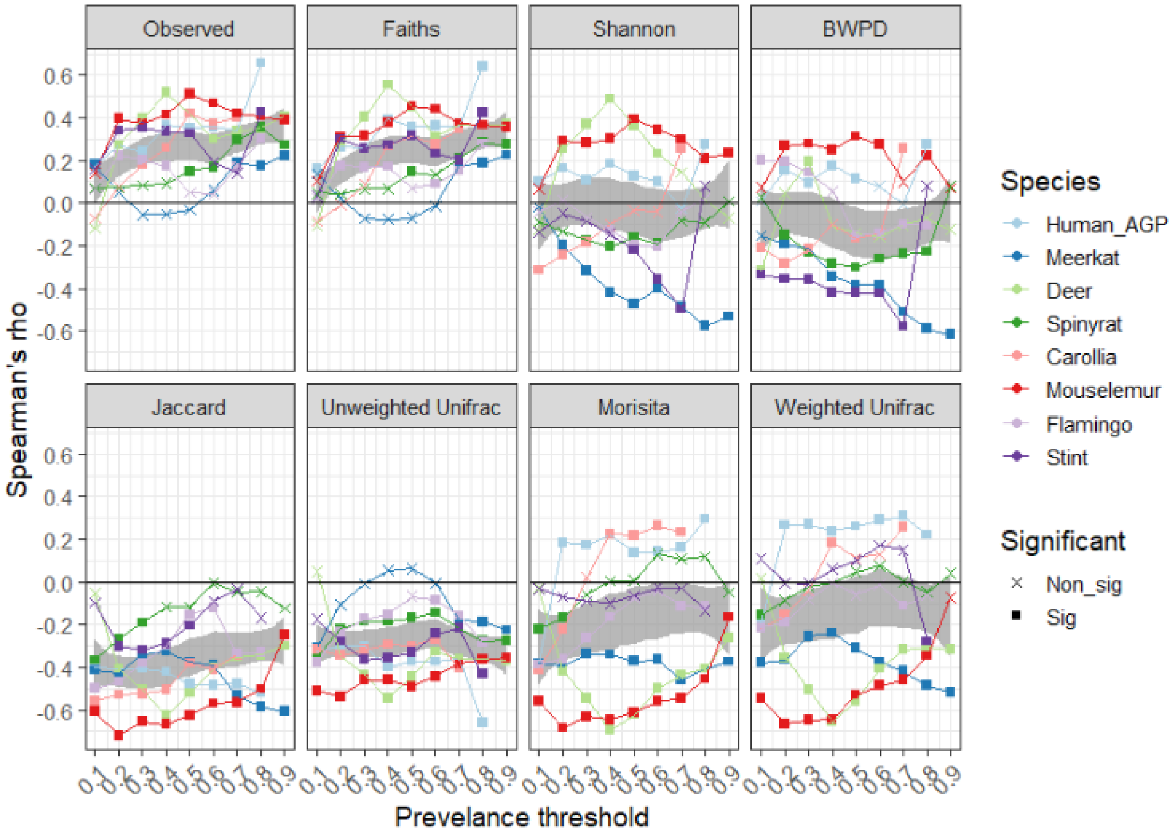

Supplement: Supplementary file 1 [file Image_1.pdf]
