# Supplementary material for: Phylogeny- and Abundance-Based Metrics Allow for the Consistent Comparison of Core Gut Microbiome Diversity Indices Across Host Species
Source: Front Microbiol. 2021 May 11;12:659918. doi: 10.3389/fmicb.2021.659918 (PMC8144293; doi:10.3389/fmicb.2021.659918)
Supplement: Supplementary file 2 [file Data_Sheet_1.PDF]

Core microbiome analysis

Alice Risely

15/03/2021

Contents

Manuscript background 2
Load packages 2
Import and view 16S gut microbiome datasets 3
Figure 1a: ASV accumulation curves with sequencing depth 4
Figure 1b: ASV accumulation curves with sample size 5
Figure 1c: Accumulated proportion of ASV detected with sample size 8
Figure 1d: Effect of sample size on regional ASV pool 10
Estimating prevalence and abundance of ASVs 11
Figure 1e: Proportion of ASVs per dataset found in 1-8 samples 13
Figure 1f: Proportion of ASV per sample detected in 1-8 samples 15
Fig 1g: Occupancy-abundance curves 18
Figure 1h: Rank abundance curves 19
Figure 1 final 21
Calculate alpha diversity per sample 21
Calculate beta dissimilarity per sample 23
Figure 2a-d: Relationship between alpha diversity and prevalence threshold 24
Figure 2e-h: Relationship between beta dissimilarity and prevalence threshold 29
Bartlett's Test for changes in variance 31
Figure 3: Mean changes to alpha and beta diversity with prevalence threshold 32
Figure S1: Correlation between sample read depth and diversity 34
Visualise diversity correlation matrix with ggparirs 36
Correlation between core diversity and unfiltered diversity 44
Figure 4: Spearman's correlation with unfiltered data 46
Mean Spearman's correlation per measure and threshold 48

Manuscript background

Analysis for the manuscript:

Phylogeny- and abundance-based diversity metrics allow for the consistent comparison of core gut microbiome diversity indices across host species

Risely, A., Gillingham, M.A.F. , Béchet, A., Brändel, S., Heni, A.C. 1, Heurich, M., Menke, S., Manser, M., Tschapka, M., Wasimuddin, & Sommer, S.

Abstract The filtering of gut microbial datasets to retain high prevalence taxa is often performed to identify a common core gut microbiome that may be important for host biological functions. However, prevalence thresholds used to identify a common core are highly variable, and it remains unclear how they affect diversity estimates and whether insights stemming from core microbiomes are comparable across studies. We hypothesized that if macroecological patterns in gut microbiome prevalence and abundance are similar across host species, then we would expect that increasing prevalence thresholds would yield similar changes to alpha diversity and beta dissimilarity scores across host species datasets. We analysed eight gut microbiome datasets based on 16S rRNA gene amplicon sequencing and collected from different host species to 1) compare macroecological patterns across datasets, including amplicon sequence variant (ASV) detection rate with sequencing depth and sample size, occupancy-abundance curves, and rank-abundance curves; 2) test whether increasing prevalence thresholds generate universal or host-species specific effects on alpha and beta diversity scores; and 3) test whether diversity scores from prevalence-filtered core communities correlate with unfiltered data. We found that gut microbiomes collected from diverse hosts demonstrated similar ASV detection rates with sequencing depth, yet required different sample sizes to sufficiently capture rare ASVs across the host population. This suggests that sample size rather than sequencing depth tends to limit the ability of studies to detect rare ASVs across the host population. Despite differences in the distribution and detection of rare ASVs, microbiomes exhibited similar occupancy-abundance and rank-abundance curves. Consequently, increasing prevalence thresholds generated remarkably similar trends in standardized alpha diversity and beta dissimilarity across species datasets until high thresholds above 70%. At this point, diversity scores tended to become unpredictable for some diversity measures. Moreover, high prevalence thresholds tended to generate diversity scores that correlated poorly with the original unfiltered data. Overall, we recommend that high prevalence thresholds over 70% are avoided, and promote the use of diversity measures that account for phylogeny and abundance (Balance-weighted phylogenetic diversity and Weighted Unifrac for alpha and beta diversity, respectively), because we show that these measures are insensitive to prevalence filtering and therefore allow for the consistent comparison of core gut microbiomes across studies without the need for prevalence filtering.

Load packages

```
library(phyloseq)
library(ggplot2)
library(plyr)
library(metagMisc)
library(tidyr)
library(RColorBrewer)
library(ggpubr)
library(dplyr)
library(vegan)
library(GGally)
library(viridis)
```

Import and view 16S gut microbiome datasets

- This is a list of phyloseq object per species.
- Each dataset is the result of DADA2 processing, with non-bacterial, mitochondrial, chloroplast, and completely unidentified (to phyla level) ASVs filtered out.
- These data are not normalised.

```r
phylo_list<-readRDS("phylo_list_unrarefied_frontiers.RDS")

phylo_list # View data
```

```
## $Human_AGP
## phyloseq-class experiment-level object
## otu_table()   OTU Table:         [ 10017 taxa and 500 samples ]
## sample_data() Sample Data:       [ 500 samples by 56 sample variables ]
## tax_table()   Taxonomy Table:    [ 10017 taxa by 7 taxonomic ranks ]
## phy_tree()    Phylogenetic Tree: [ 10017 tips and 10015 internal nodes ]
##
## $Meerkat
## phyloseq-class experiment-level object
## otu_table()   OTU Table:         [ 4475 taxa and 137 samples ]
## sample_data() Sample Data:       [ 137 samples by 49 sample variables ]
## tax_table()   Taxonomy Table:    [ 4475 taxa by 7 taxonomic ranks ]
## phy_tree()    Phylogenetic Tree: [ 4475 tips and 4474 internal nodes ]
##
## $Deer
## phyloseq-class experiment-level object
## otu_table()   OTU Table:         [ 10450 taxa and 136 samples ]
## sample_data() Sample Data:       [ 136 samples by 9 sample variables ]
## tax_table()   Taxonomy Table:    [ 10450 taxa by 7 taxonomic ranks ]
## phy_tree()    Phylogenetic Tree: [ 10450 tips and 10449 internal nodes ]
##
## $Spinyrat
## phyloseq-class experiment-level object
## otu_table()   OTU Table:         [ 6109 taxa and 196 samples ]
## sample_data() Sample Data:       [ 196 samples by 8 sample variables ]
## tax_table()   Taxonomy Table:    [ 6109 taxa by 7 taxonomic ranks ]
## phy_tree()    Phylogenetic Tree: [ 6109 tips and 6108 internal nodes ]
##
## $Carollia
## phyloseq-class experiment-level object
## otu_table()   OTU Table:         [ 11545 taxa and 169 samples ]
## sample_data() Sample Data:       [ 169 samples by 8 sample variables ]
## tax_table()   Taxonomy Table:    [ 11545 taxa by 7 taxonomic ranks ]
## phy_tree()    Phylogenetic Tree: [ 11545 tips and 11544 internal nodes ]
##
## $Mouselemur
## phyloseq-class experiment-level object
## otu_table()   OTU Table:         [ 2251 taxa and 182 samples ]
## sample_data() Sample Data:       [ 182 samples by 40 sample variables ]
## tax_table()   Taxonomy Table:    [ 2251 taxa by 7 taxonomic ranks ]
## phy_tree()    Phylogenetic Tree: [ 2251 tips and 2250 internal nodes ]
##
```

```
## $Flamingo
## phyloseq-class experiment-level object
## otu_table()   OTU Table:         [ 8306 taxa and 552 samples ]
## sample_data() Sample Data:       [ 552 samples by 23 sample variables ]
## tax_table()   Taxonomy Table:    [ 8306 taxa by 7 taxonomic ranks ]
## phy_tree()    Phylogenetic Tree: [ 8306 tips and 8305 internal nodes ]
##
## $Stint
## phyloseq-class experiment-level object
## otu_table()   OTU Table:         [ 9545 taxa and 98 samples ]
## sample_data() Sample Data:       [ 98 samples by 22 sample variables ]
## tax_table()   Taxonomy Table:    [ 9545 taxa by 7 taxonomic ranks ]
## phy_tree()    Phylogenetic Tree: [ 9545 tips and 9544 internal nodes ]
```

Figure 1a: ASV accumulation curves with sequencing depth

- Here we use the package ranacapa to generate rarefaction curves per sample per species dataset.
- Loop through phyloseq objects to generate each rarefaction fig.

```
rarefaction_fig<-list() #make an empty list
uniq <- names(phylo_list) # make a list of species to subset sequentially

## colour palette
palette<-brewer.pal(10,"Paired")
palette<-palette[c(-7,-8)]

for (i in 1:length(uniq)){ #for species i

  data_1<-phylo_list[[i]] #subset the phyloseq object for species i
  data_1<-prune_taxa(taxa_sums(data_1)>0, data_1)  #remove any traces of
  # taxa that are no longer present in dataset


p <- ranacapa::ggrare(data_1, step = 100,   se = FALSE, plot = FALSE)+
  xlim(c(0,30000)) # limit x axis to 30,000 reads per sample

p + xlim(c(0,30000))


p1<- p +geom_line(col = palette[[i]], size = 0.1)+
  geom_vline(xintercept=10000)+
  geom_hline(yintercept=200, linetype = "dashed", size = 0.5)+
  theme(legend.position = "none")+
  theme_bw(base_size = 11)+
  theme(axis.text.x = element_blank(), axis.title.y = element_blank())+
  xlab(print(uniq[[i]]))+
  theme(plot.margin=unit(c(0.2,0.2,0.2,0.2),"cm"))

rarefaction_fig[[i]]<-p1

}
```

```
Fig1a<-ggarrange(rarefaction_fig[[1]],rarefaction_fig[[2]],rarefaction_fig[[3]],
  rarefaction_fig[[4]], rarefaction_fig[[5]], rarefaction_fig[[6]],
  rarefaction_fig[[7]], rarefaction_fig[[8]], ncol = 4, nrow = 2)

annotate_figure(Fig1a, bottom = text_grob("Sequencing depth", size = 14),
  left = text_grob("Detected ASVs", rot = 90, size = 14), top =
    text_grob("Figure 1a", size = 14))
```

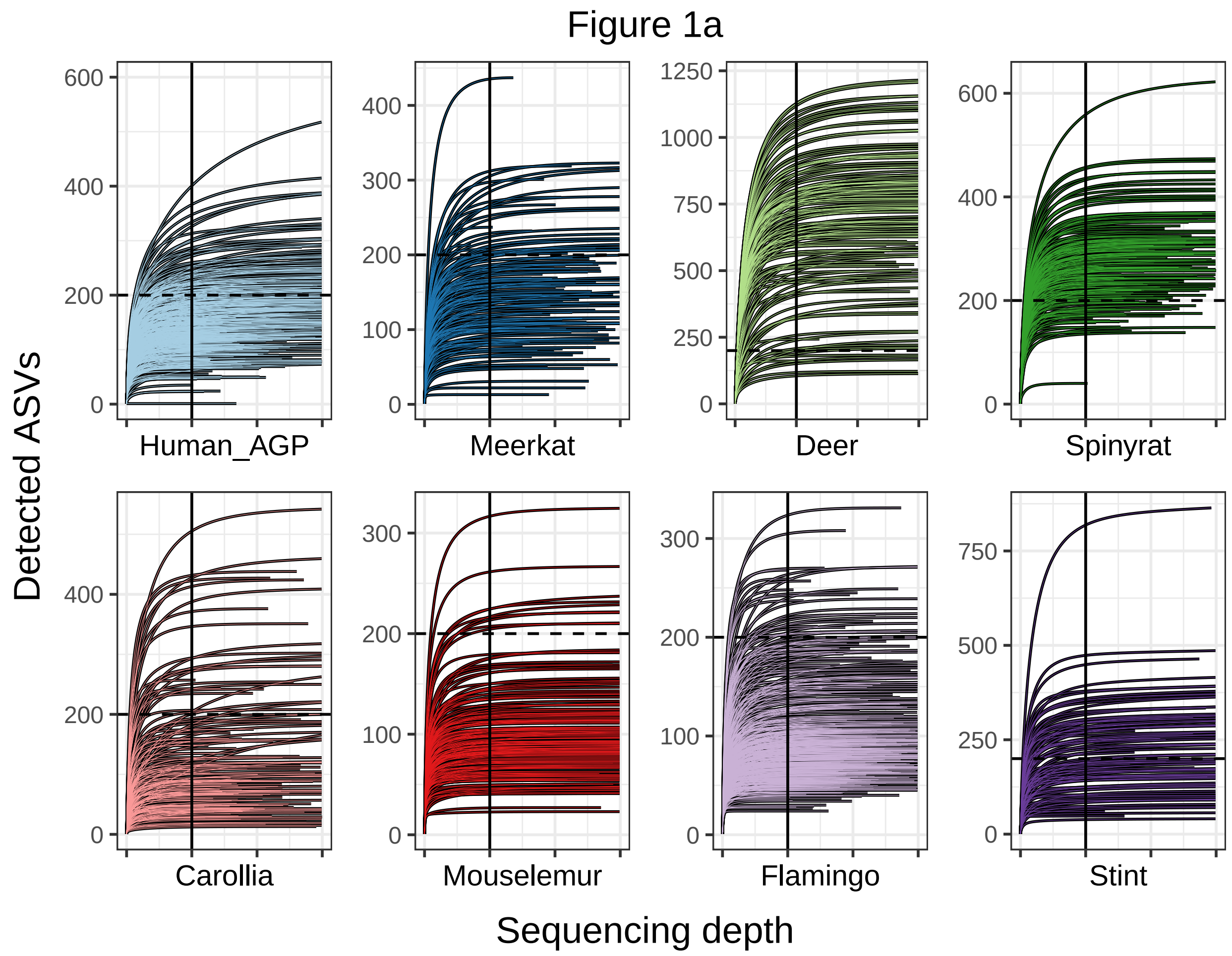


```
Fig1a<- annotate_figure(Fig1a, bottom = text_grob("Sequencing depth", size = 14),
  left = text_grob("Detected ASVs", rot = 90, size = 14))
```

Figure 1b: ASV accumulation curves with sample size

- Here we use vegans specaccum() function to generate ASV accumulation curves per dataset.
- Predict regional ASV pool using specpool() function.
- Calculate accumulating effect of sample size on regional ASV pool prediction using poolaccum() function.

```
SAClist<-list() #make an empty list for specaccum() results
poolaccum_list<-list() #make an empty list for poolaccum() resuls
```

```r
uniq <- names(phylo_list) # make a list of species to subset sequentially

for (i in 1:length(uniq)){ #for species i

  data_1<-phylo_list[[i]] #subset the phyloseq object for species i
    #remove any traces of taxa that are no longer present in dataset
  data_1<-prune_taxa(taxa_sums(data_1)>0, data_1)
  #transpose the OTU table
  data_1_matrix<-data.frame(t(data.frame(otu_table(data_1))))
  data_1_specaccum<-vegan::specaccum(data_1_matrix, method="random",
    permutations = 999) #apply specaccum()
  ## the output is in list form, so we need to make this into a dataframe
  sac_df<- data_1_specaccum$sites ##sites = samples
  sac_df<-data.frame(sac_df)
  names(sac_df)[1]<-"Site"
  sac_df$Richness <-  data_1_specaccum$richness #import ASV richness to dataframe
  sac_df$SD <-  data_1_specaccum$sd #import the standard deviation


  ## this next step estimates the TOTAL number of ASVs in the ASV pool.
  sac_total_estimated<-vegan::specpool(data_1_matrix)
  sac_df$Total <- sac_total_estimated$boot ##add this to our dataframe
  sac_df$Total_jackknife <- sac_total_estimated$jack1 ## using jackknife method
  sac_df$Total_chao <- sac_total_estimated$chao ## using jackknife method
  sac_df$Species <- as.character(uniq[[i]]) #add species name,
  #for when we combine dataframes for all species
  SAClist[[i]]<-sac_df #add this dataframe as an element in the empty list
  #and repeat for the next species


  ### check affect of sample size on total estimate
  poolaccum_df<-poolaccum(data_1_matrix, permutations = 100, minsize = 3)
  poolaccum_df_means<- data.frame(poolaccum_df$means)
  poolaccum_df_means$Species<-uniq[[i]]
  poolaccum_list[[i]]<-  poolaccum_df_means

}

# Rbind into two dataframes to generate figs 1b-d

names(SAClist)<-uniq #name elements of the list by species
sac_df_all<- do.call(rbind, SAClist) #rbind all our 8 dataframes together#rbind all our 8 dataframes to

poolaccum_df_all<-do.call(rbind, poolaccum_list) #rbind all our 8
#dataframes together
poolaccum_df_all$Species<-factor(poolaccum_df_all$Species, levels = uniq)
```

- Use jackknife 1 method for predicting regional ASV pool (other options are bootstrapping and chao1).
- Format for figures.

```r
## format for figures

#subset just the eight distinct estimated totals per species
```

```r
species_totals<-sac_df_all %>% distinct(Total_jackknife, .keep_all = T)
species_totals[,1]<-700 #here we put 700 just because we want a number that is
#larger than the largest samples size (flamingo)
species_totals[,2]<-species_totals$Total_jackknife
species_totals[,3]<-NA
species_totals[,4]<-NA

sac_df_fig<-rbind(sac_df_all, species_totals) #combine
sac_df_fig$Species<-factor(sac_df_fig$Species, levels = uniq)

## colour palette
palette<-brewer.pal(10,"Paired")
palette<-palette[c(-7,-8)]
#show_col(palette)

#Fig1b

ggplot(sac_df_fig, aes(x = Site, y = Richness, group = Species))+
  geom_line(alpha=1, linetype = "dashed", size = 1, aes(col = Species))+
  geom_point( aes(col=Species), size = 1.5, alpha = 0.7)+  theme_bw()+
  xlab("N")+
  ylab("Number of ASVs")+
  theme(text=element_text(size=14))+
  scale_color_manual(values = palette)+
  ggtitle("Figure 1b")+
  guides(colour = guide_legend(override.aes = list(size=5)))
```

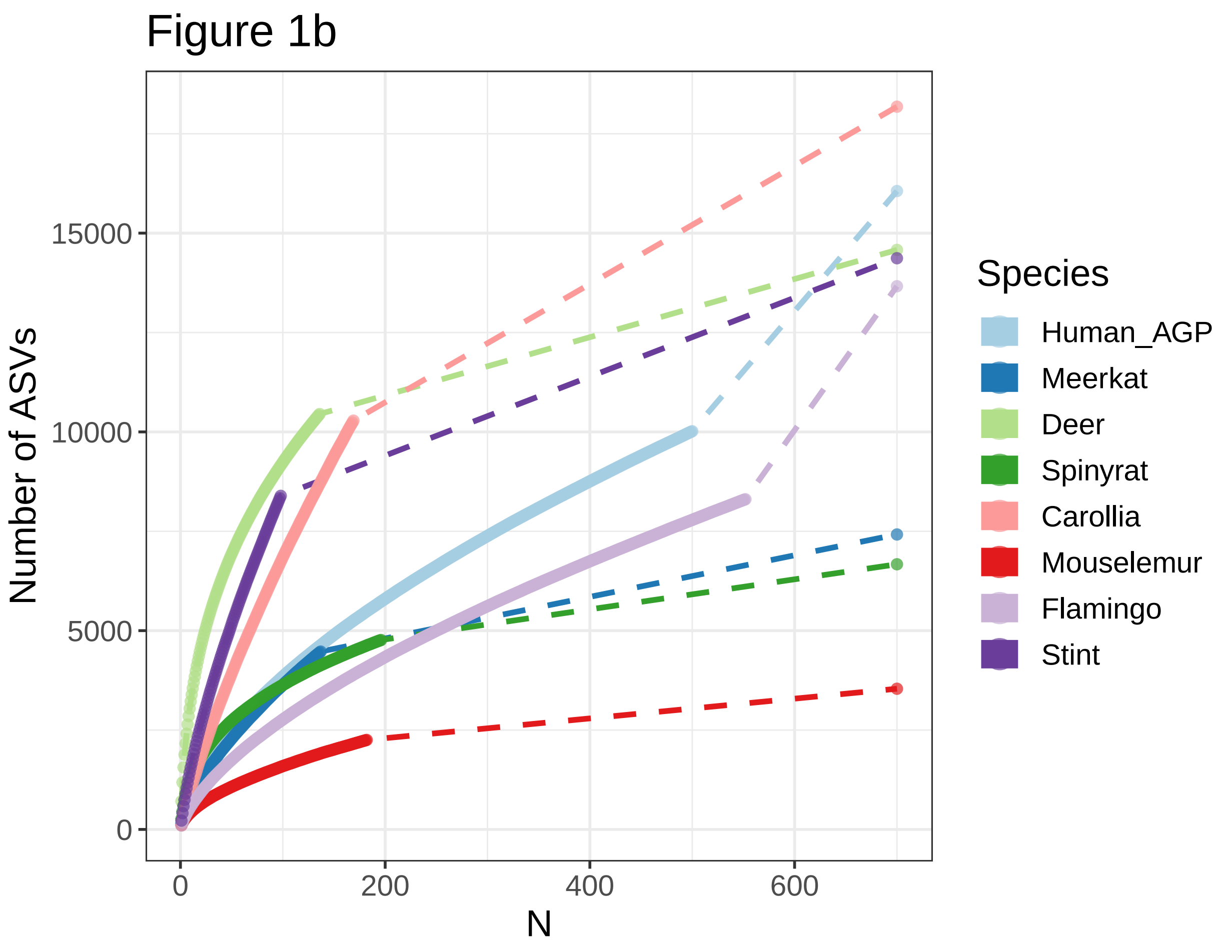


```
Fig1b<-ggplot(sac_df_fig, aes(x = Site, y = Richness, group = Species))+
  geom_line(alpha=1, linetype = "dotted", size = 1, aes(col = Species))+
  geom_point( aes(col=Species), size = 1.5, alpha = 1)+
  theme_bw()+
  xlab("N")+
  ylab("Number of ASVs")+
  theme(text=element_text(size=14))+
  theme(legend.position = "none")+
  scale_color_manual(values = palette)+
  guides(colour = guide_legend(override.aes = list(size=5)))
```

Figure 1c: Accumulated proportion of ASV detected with sample size

```
### summarise proportion ASVs detected in comparison to total number predicted to be within
#the regional species pool

summary<-sac_df_all %>% group_by(Species) %>%
  summarize(max_asvs = max(Richness), predicted = max(Total_jackknife))

summary$percent<-summary$max_asvs/summary$predicted
```

```
sac_df_all$Percent<-sac_df_all$Richness/sac_df_all$Total_jackknife
sac_df_all$Species<-factor(sac_df_all$Species, levels = uniq)
segments_df<-subset(sac_df_all, Percent <0.51 & Percent > 0.49)
segments_df<-segments_df%>%distinct(Species, .keep_all =TRUE)


ggplot(sac_df_all, aes(x = Site, y = Percent, group = Species))+
  geom_line(alpha=0.5, linetype = "dashed")+
  geom_point( aes(col=Species), size = 1, alpha = 0.9)+
  theme_bw()+
  xlab("N")+
  ylab("Proportion ASVs detected")+
 ylim(0,1)+
  theme(text=element_text(size=14))+
  scale_color_manual(values = palette)+
  geom_hline(yintercept = 0.5, linetype = "dashed")+
 guides(colour = guide_legend(override.aes = list(size=7)))+
  ggtitle("Figure 1c")+
   geom_segment(data = segments_df, aes(x = Site, xend = Site, y = 0,
     yend =0.5, col = Species), size = 1, linetype = "dashed")
```

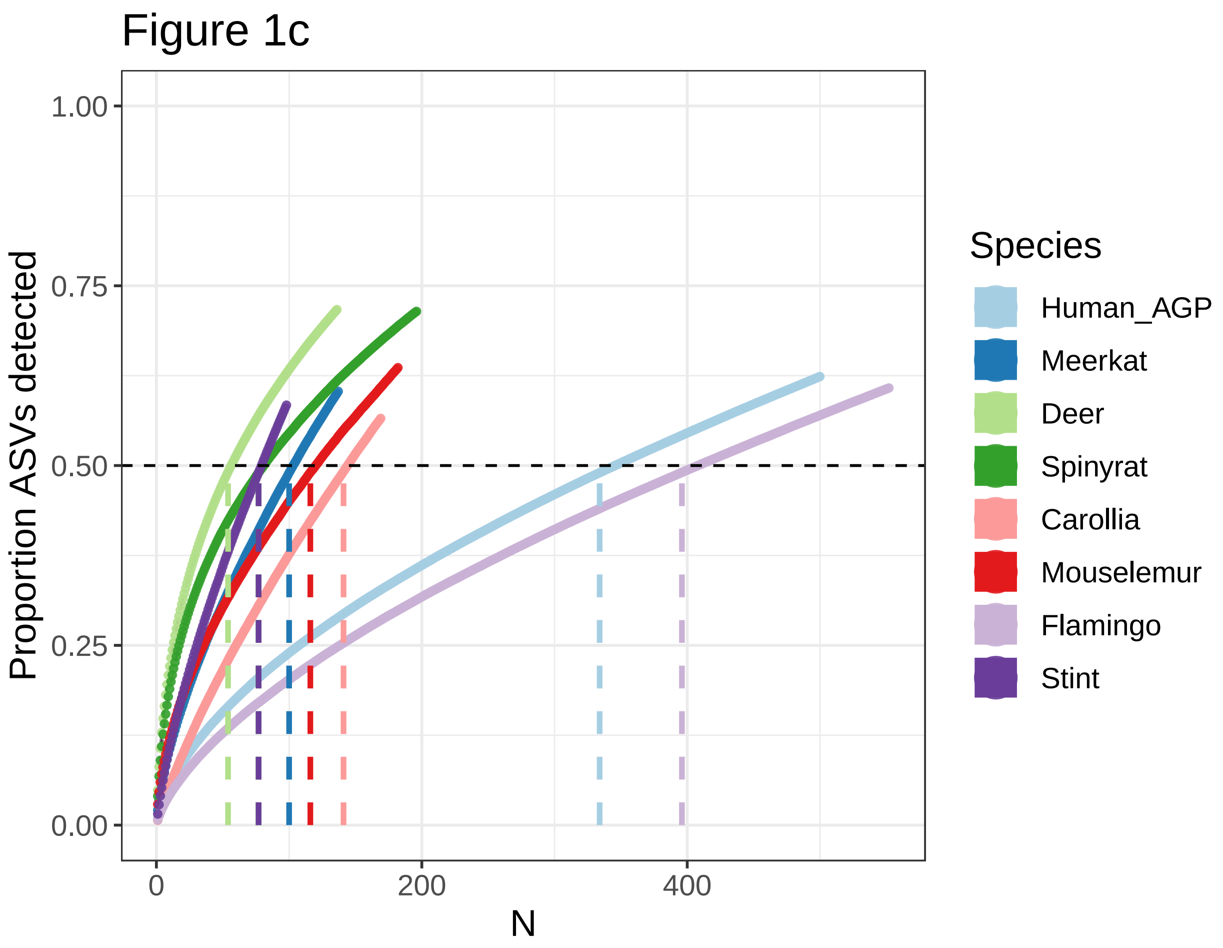

```r
Fig1c <- ggplot(sac_df_all, aes(x = Site, y = Percent, group = Species))+
  geom_line(alpha=0.5, linetype = "dashed")+
  geom_point( aes(col=Species), size = 1, alpha = 0.9)+
  theme_bw()+
  xlab("N")+
  ylab("Proportion ASVs detected")+
  ylim(0,1)+
  theme(text=element_text(size=14))+
  theme(legend.position = "none")+
  scale_color_manual(values = palette)+
  geom_hline(yintercept = 0.5, linetype = "dashed")+
  guides(colour = guide_legend(override.aes = list(size=7)))+
  geom_segment(data = segments_df, aes(x = Site, xend = Site, y = 0,
    yend =0.5, col = Species), size = 1, linetype = "dotted")
```

Figure 1d: Effect of sample size on regional ASV pool

```r
## use poolaccum_df_all df created earlier

max_estimates<-poolaccum_df_all %>% group_by(Species) %>%
  summarise(max_est = max(Jackknife.1), max_n = max(N))

ggplot(poolaccum_df_all, aes(x = N, y = Jackknife.1, group = Species))+
  geom_point(aes(col=Species), size = 1.5, alpha = 0.7)+
  scale_color_manual(values = palette)+
 # geom_hline(yintercept = max_estimates$max_est, color = c(palette), size = 1)
  geom_segment(data = max_estimates, aes(x = max_n, xend = 600, y = max_est,
    yend =max_est, col = Species), size = 1, linetype = "dashed")+
  theme_bw(base_size = 14)+
  guides(colour = guide_legend(override.aes = list(size=5)))+
  ggtitle("Figure 1d")+
  ylab("Predicted regional ASV pool")
```

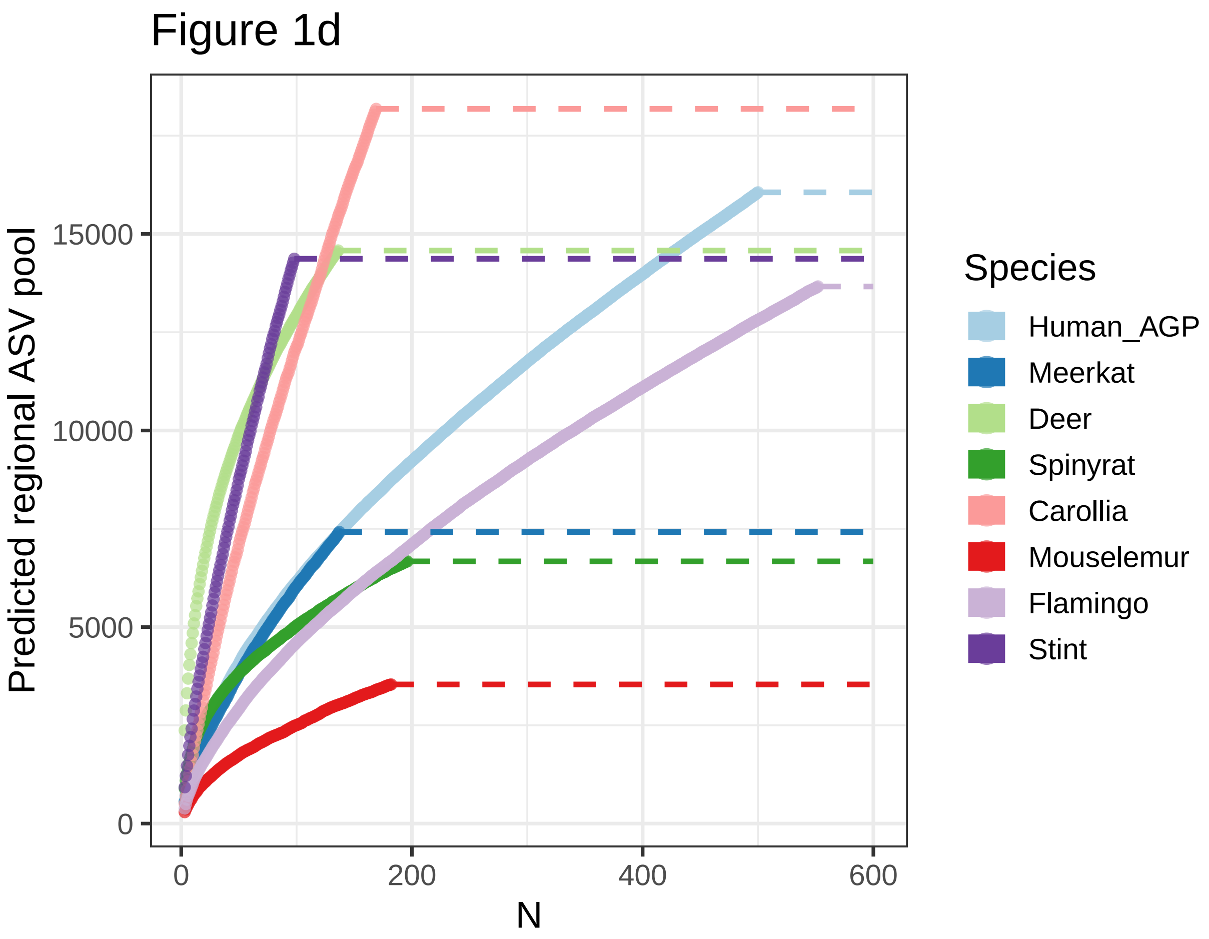


```
Fig1d<-ggplot(poolaccum_df_all, aes(x = N, y = Jackknife.1, group = Species))+
  geom_point(aes(col=Species), size = 1.5, alpha = 0.7)+
  scale_color_manual(values = palette)+
 # geom_hline(yintercept = max_estimates$max_est, color = c(palette), size = 1)
  geom_segment(data = max_estimates, aes(x = max_n, xend = 600, y = max_est,
    yend =     max_est, col = Species), size = 1, linetype = "dashed")+
  theme_bw(base_size = 14)+
  guides(colour = guide_legend(override.aes = list(size=5)))+
  ylab("Predicted regional ASV pool")


Fig1_upper<-ggarrange(Fig1a, Fig1b, Fig1c, Fig1d, ncol = 4, widths = c(2,1,0.9,1),
  common.legend = T, legend = "top", labels = c("A", "B", "C", "D"))
```

Estimating prevalence and abundance of ASVs

- Next we estimate prevalence and abundance values for every ASV per dataset.
- Generate prevalence function
- Rarefy to 10,000 reads per sample first to control for differences amongst datasets in library size (this doens't make much difference to results).

```r
## function for prevalence
prevalence <- function(physeq, add_tax = TRUE){
    ## Check if taxa are rows
  trows <- taxa_are_rows(physeq)
  ## Extract OTU table
  otutab <- as.data.frame(otu_table(physeq))
  ## Transpose OTU table (species should be arranged by rows)
  if(trows == FALSE){
    otutab <- t(otutab)
  }
  ## Estimate prevalence (number of samples with OTU present)
  prevdf <- apply(X = otutab,
                  #MARGIN = ifelse(trows, yes = 1, no = 2),
    # for a non-transposed data
                  MARGIN = 1,
                  FUN = function(x){sum(x > 0)})
  ## Add total and average read counts per OTU
  prevdf <- data.frame(Prevalence = prevdf,
                       TotalAbundance = taxa_sums(physeq),
                       MeanAbundance = rowMeans(otutab),
                       MedianAbundance = apply(otutab, 1, median))
  ## Add taxonomy table
  if(add_tax == TRUE && !is.null(tax_table(physeq, errorIfNULL = F))){
    prevdf <- cbind(prevdf, tax_table(physeq))
  }
  return(prevdf)
}
```

- Loop prevalence function through each dataset.
- Yay loops.

```r
## start loop

Prevlist<-list()
uniq <- names(phylo_list)

for (i in 1:length(uniq)){

  data_1<-phylo_list[[i]]  #for loop 1 (uniq)
  data_1<-rarefy_even_depth(data_1, sample.size = 10000, rngsee = 100, replace = TRUE,
    trimOTUs=FALSE,verbose=FALSE)
  occupancy_abundance<-prevalence(data_1)
  occupancy_abundance$host_species <- as.character(uniq[[i]])
  occupancy_abundance$RelAbundance<- (occupancy_abundance$TotalAbundance/
      sum(occupancy_abundance$TotalAbundance))
  occupancy_abundance$RelPrev<-(occupancy_abundance$Prevalence /
      length(sample_data(data_1)$feature.id))
  occupancy_abundance$ASV<-taxa_names(data_1) #this is important! Don't use
  #'row.names' as it sneakily adds a 1 at the end if it occurs twice
  occupancy_abundance$Sample_size<-length(unique(sample_data(data_1)$Sample))
  Prevlist[[i]]<-occupancy_abundance

}
```

```r
#combine
occupancy_abundance_df<-do.call(rbind, Prevlist)
occupancy_abundance_df$host_species<-factor(occupancy_abundance_df$host_species,
  levels = uniq)
```

Figure 1e: Proportion of ASVs per dataset found in 1-8 samples

- Use true prevalence rather than percentage because interested in how many ASVs occur in just one sample.

```r
## proportion of taxa occuring in just one individula

prevs<-c(0,1,2,3,4,5,6,7,8) # prevalence categories of interest

occupancy_abundance_df$Prevalence_alt<-ifelse(occupancy_abundance_df$Prevalence %in%
    prevs, occupancy_abundance_df$Prevalence, "Over_8")

proportion_singletons<-data.frame(table(occupancy_abundance_df$Prevalence_alt,
  occupancy_abundance_df$host_species))

names(proportion_singletons)<-c("Prevelance", "Species", "Count")

# remove taxa with zero prevalence (which were removed by rarefaction). This makes up
# about 10% of ASVs per dataset (but doesn't make a difference to overall patterns)

proportion_singletons<-subset(proportion_singletons, Prevelance != "0")

ggplot(proportion_singletons, aes(fill=Prevelance, y=Count, x=Species)) +
    geom_bar(position="fill", stat="identity")+
  ylab("Proportion")+
  theme_grey(base_size = 12)+
  scale_fill_viridis(discrete = TRUE, option = "D")+
  scale_y_continuous(breaks = c(0,0.1,0.2,0.3,0.4,0.5,0.6,0.7,0.8,0.9, 1.0))+
 theme(axis.title.x = element_blank())+
  theme(axis.text.x = element_text(angle =35, hjust = 1))
```

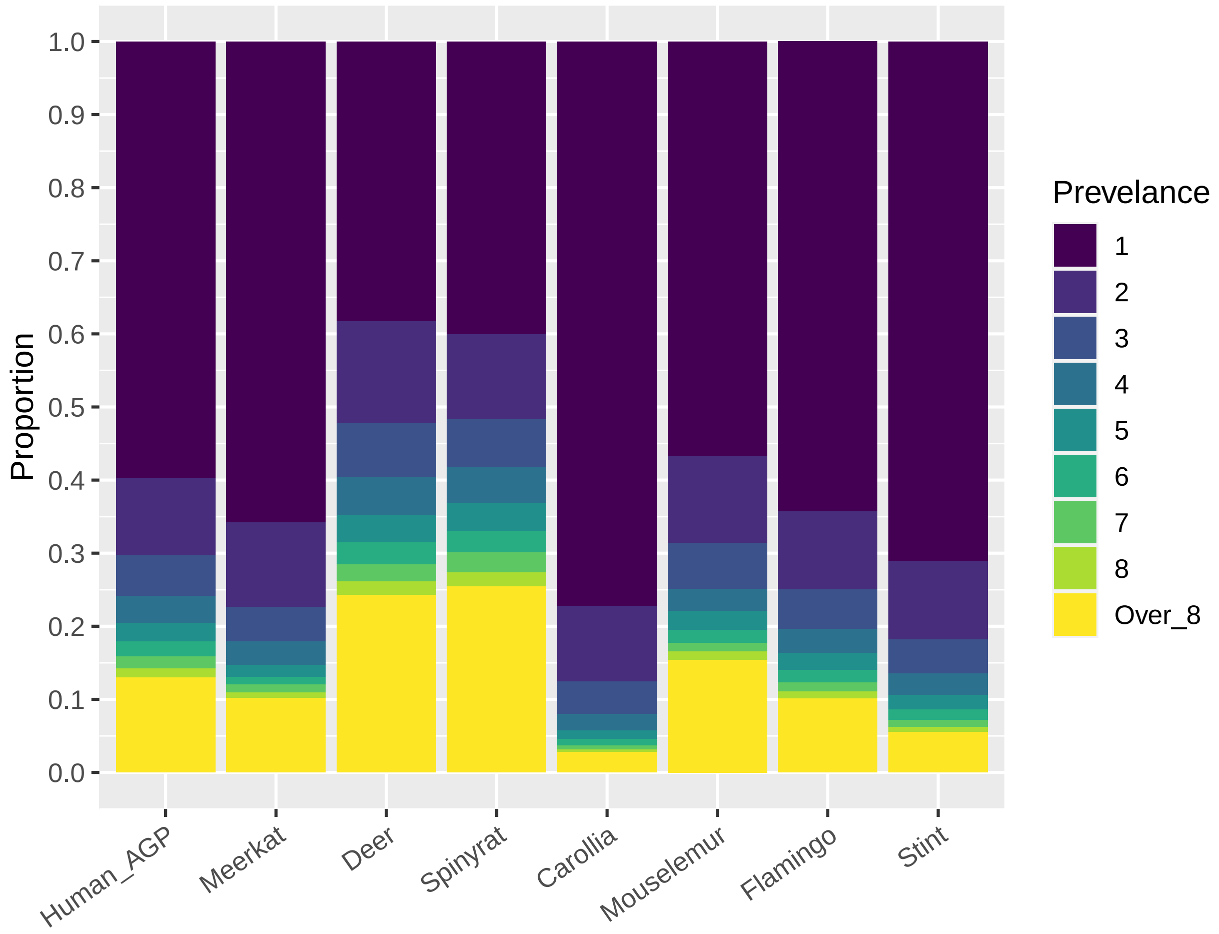


```r
Fig1e<-ggplot(proportion_singletons, aes(fill=Prevelance, y=Count, x=Species)) +
    geom_bar(position="fill", stat="identity")+
 # scale_fill_brewer("Diamond\nclarity", direction = -1)+
  ylab("Proportion")+
  theme_minimal(base_size = 12)+
  scale_fill_viridis(discrete = TRUE, option = "D")+
  scale_y_continuous(breaks = c(0,0.1,0.2,0.3,0.4,0.5,0.6,0.7,0.8,0.9, 1.0))+
 theme(axis.title.x = element_blank(), axis.text.x = element_blank())+
  theme(legend.position = "none")

head(proportion_singletons)
```

```
##   Prevelance   Species Count
## 2          1 Human_AGP  5454
## 3          2 Human_AGP   965
## 4          3 Human_AGP   506
## 5          4 Human_AGP   338
## 6          5 Human_AGP   230
## 7          6 Human_AGP   190
```

```r
paste("The proportion of ASVs that occurred in just one sample was",
  sum(subset(proportion_singletons, Prevelance=="1")$Count)/sum(proportion_singletons$Count))
```

```
## [1] "The proportion of ASVs that occurred in just one sample was 0.599028543360803"
```

Figure 1f: Proportion of ASV per sample detected in 1-8 samples

```r
fig_list<-list()
prevalence_barplot_df<-list()

for (i in 1:length(uniq)){ #for species i

  data_1<-phylo_list[[i]] #subset the phyloseq object for species i
  data_1<-prune_taxa(taxa_sums(data_1)>0, data_1)

  ## add prevalence category to taxtable as column
  taxtable<-data.frame(data_1@tax_table@.Data)
  taxtable$ASV<-row.names(taxtable)
  oc_df<-subset(occupancy_abundance_df, host_species == uniq[i])
  oc_df<-oc_df[,c("ASV", "Prevalence_alt")]
  new_taxtable<-merge(taxtable, oc_df, by = "ASV")
  row.names(new_taxtable)<-new_taxtable$ASV
  new_taxtable<-new_taxtable[row.names(taxtable),]
  new_taxtable<- new_taxtable[,c("Kingdom","Prevalence_alt")]
  new_taxtable<-tax_table(new_taxtable)
  taxa_names(new_taxtable)<-row.names(taxtable)
  tax_table(data_1) <- new_taxtable


  # make presence-absence so that abundance == prevalence
 data_pa<- phyloseq_standardize_otu_abundance(data_1, method = "pa")
 max(otu_table(data_pa))
 data_glom<-tax_glom(data_pa, taxrank = "ta2") #ta2 is the prevalence category
taxa_names(data_glom)
tax_table(data_glom)
taxa_names(data_glom)<-as.character(data.frame(tax_table(data_glom))$ta2)
 # now otu table represents numbers of ASVs in each prevalence category

 ps_data1<-psmelt(data_glom) # melt dataframe
 ps_data1<-ps_data1[,c("OTU", "Sample", "Abundance", "ta2")]
 names(ps_data1)<-c("ASV", "Sample", "Abundance", "Prev_cat")
 ps_data1$Species<-uniq[i]

 prevalence_barplot_df[[i]]<-ps_data1

fig<-ps_data1 %>%
  ggplot(aes(x = Sample, y = Abundance, fill = Prev_cat)) +
  geom_bar(stat = "identity", width = 1, position = "fill")+
   scale_fill_viridis(discrete = TRUE, option = "D")+
  ggtitle(uniq[i])
```

```
fig_list[[i]]<-fig

}

# Figure by sample

ggarrange(fig_list[[1]], fig_list[[2]],fig_list[[3]],fig_list[[4]],
  fig_list[[5]],fig_list[[6]],fig_list[[7]],fig_list[[8]], ncol = 4, nrow = 2,
  common.legend = TRUE, legend = "bottom")
```

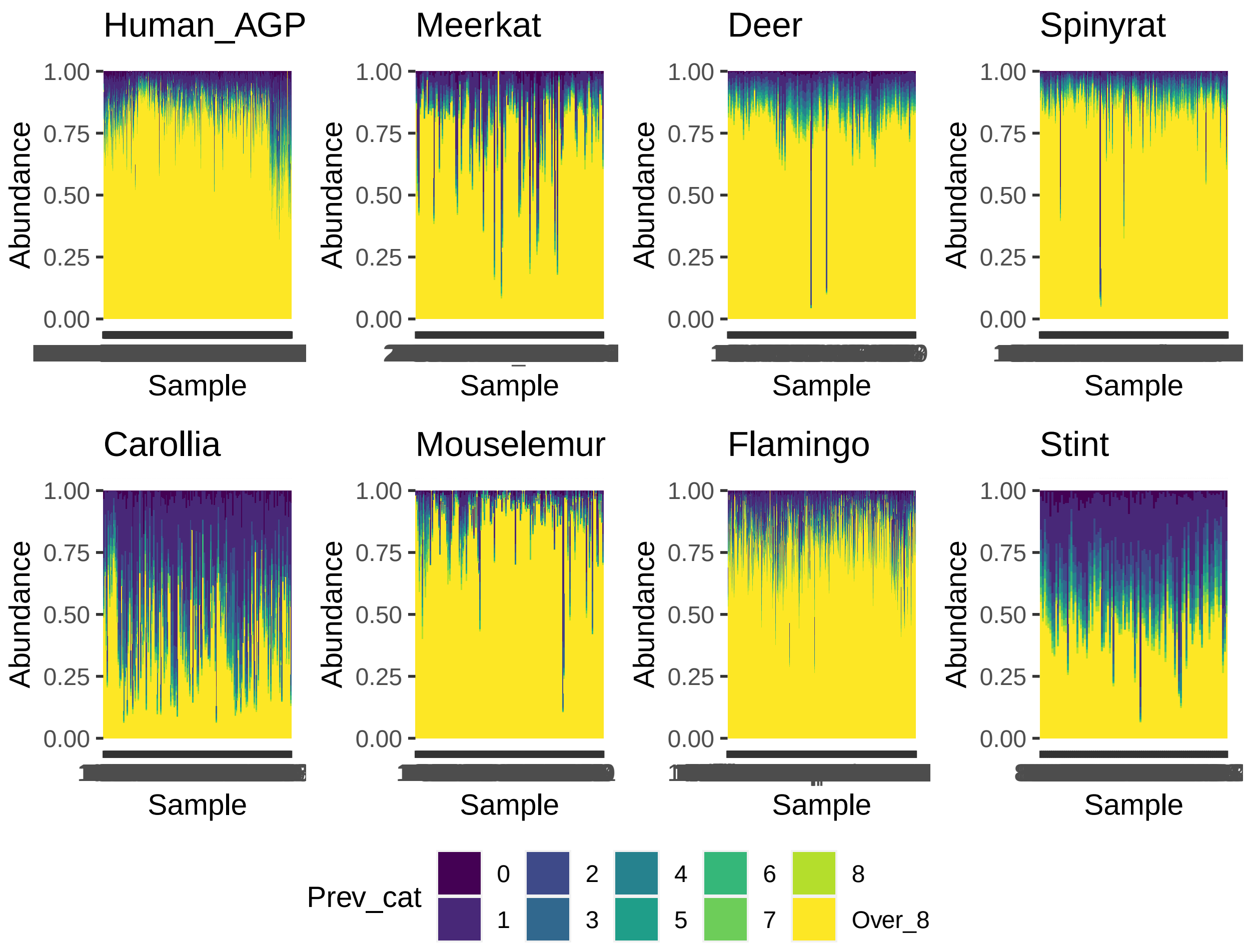


```
prevalence_barplot_df<-do.call(rbind,prevalence_barplot_df)
prevalence_barplot_df$Species<-factor(prevalence_barplot_df$Species, levels = uniq)
prevalence_barplot_df<-subset(prevalence_barplot_df, Prev_cat != "0") # remove taxa
#with zero prevalence (which were removed by rarefaction)


ggplot(prevalence_barplot_df, aes(fill=Prev_cat, y=Abundance, x=Species)) +
    geom_bar(position="fill", stat="identity")+
  ylab("Proportion")+
  theme_grey(base_size = 12)+
  scale_fill_viridis(discrete = TRUE, option = "D")+
```

```
  scale_y_continuous(breaks = c(0,0.1,0.2,0.3,0.4,0.5,0.6,0.7,0.8,0.9, 1.0))+
  guides(fill=guide_legend(title="Prevalence"))
```

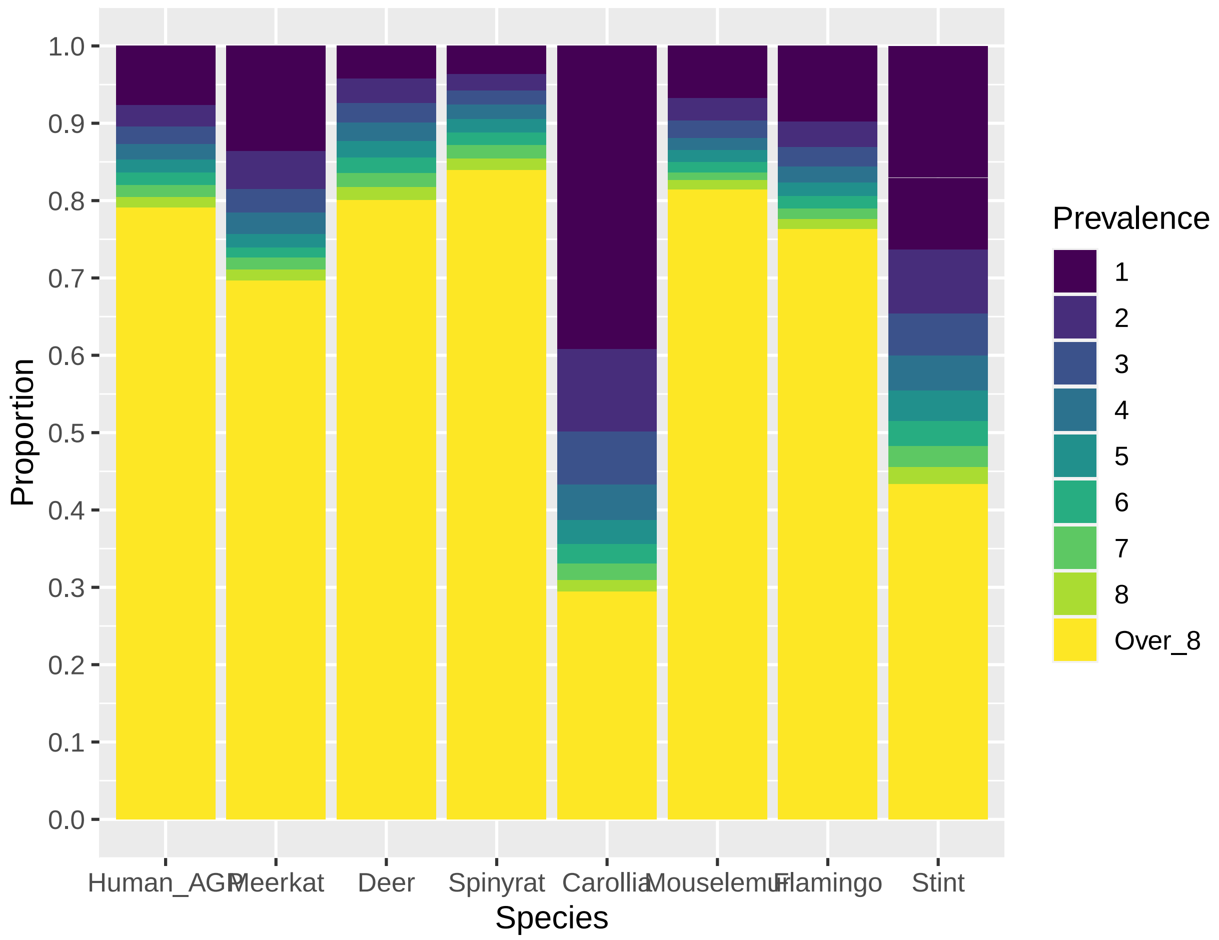


```
Fig1f<-ggplot(prevalence_barplot_df, aes(fill=Prev_cat, y=Abundance, x=Species)) +
    geom_bar(position="fill", stat="identity")+
  ylab("Proportion")+
  theme_minimal(base_size = 12)+
  scale_fill_viridis(discrete = TRUE, option = "D")+
  scale_y_continuous(breaks = c(0,0.1,0.2,0.3,0.4,0.5,0.6,0.7,0.8,0.9, 1.0))+
 theme(axis.title.x = element_blank(), axis.text.x = element_blank())+
 theme(axis.title.y = element_blank(), axis.text.y = element_blank())+
  guides(fill=guide_legend(title="Prevalence"))

count_all<-prevalence_barplot_df %>% group_by(Species) %>%
  summarise(sum = sum(Abundance))

count_singltons<-prevalence_barplot_df %>% subset(Prev_cat == "1") %>%
  group_by(Species) %>%
  summarise(sum = sum(Abundance))

count_singltons$sum/count_all$sum
```

```
## [1] 0.07656006 0.13634783 0.04239296 0.03663052 0.39233756 0.06764658 0.09791643
## [8] 0.26327083
```

```r
mean(count_singltons$sum[c(-5, -8)]/count_all$sum[c(-5, -8)])
```

```
## [1] 0.07624906
```

```r
paste("The proportion of ASVs that occurred in just one sample was", sum(subset(prevalence_barplot_df, [illegible]
```

```
## [1] "The proportion of ASVs that occurred in just one sample was 0.0960853691517173"
```

Fig 1g: Occupancy-abundance curves

```r
ggplot(occupancy_abundance_df, aes(y = RelPrev, x = RelAbundance, group = host_species,
  col = host_species))+
  geom_smooth(size = 2)+
  theme_bw(base_size = 14)+
  scale_x_log10()+
  scale_color_manual(values = palette)+
  xlab("Relative abundance")+
  ylab("Prevalence")+
  theme(legend.position = "none")+
  ggtitle("Figure 1g")
```

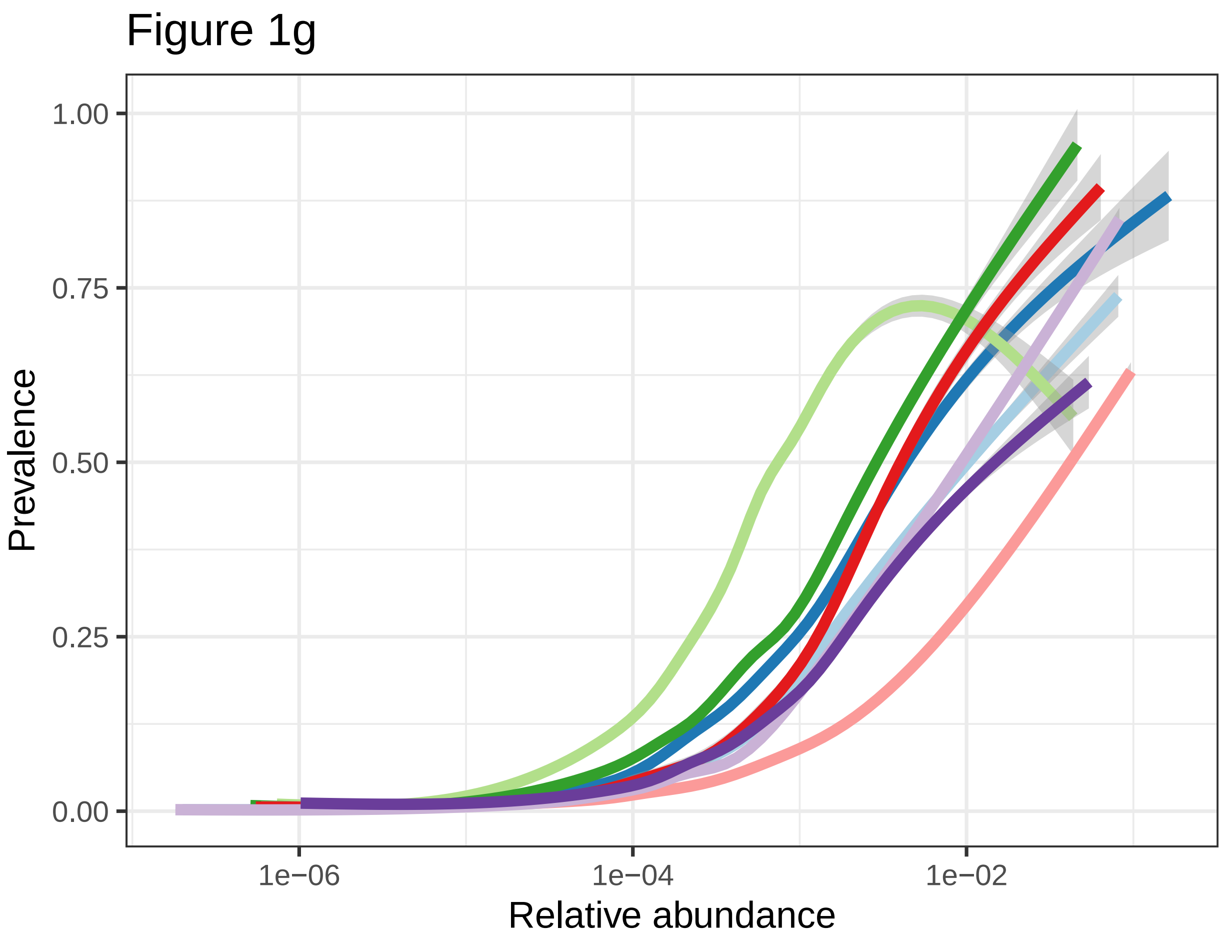


```r
Fig1g<-ggplot(occupancy_abundance_df, aes(y = RelPrev, x = RelAbundance,
  group = host_species, col = host_species))+
  geom_smooth(size = 2)+
  theme_bw(base_size = 14)+
  scale_x_log10()+
  scale_color_manual(values = palette)+
  xlab("Relative abundance")+
  ylab("Prevalence")+
  theme(legend.position = "none")
```

Figure 1h: Rank abundance curves

```r
## add abundance rank as column

occupancy_abundance_df<-occupancy_abundance_df %>%
  arrange(host_species, -RelAbundance)%>%
  group_by(host_species) %>%
  mutate(Rank = rank(-RelAbundance))

ggplot(occupancy_abundance_df, aes(y = RelAbundance, x = Rank, group = host_species,
```

```
  col = host_species))+
  geom_smooth(size = 2)+
  theme_bw(base_size = 14)+
  scale_x_log10(limits = c(1,1000))+
  scale_color_manual(values = palette)+
  ylab("Relative abundance")+
  xlab("Abundance rank")+
  theme(legend.position = "none")+
  ggtitle("Figure 1h")
```

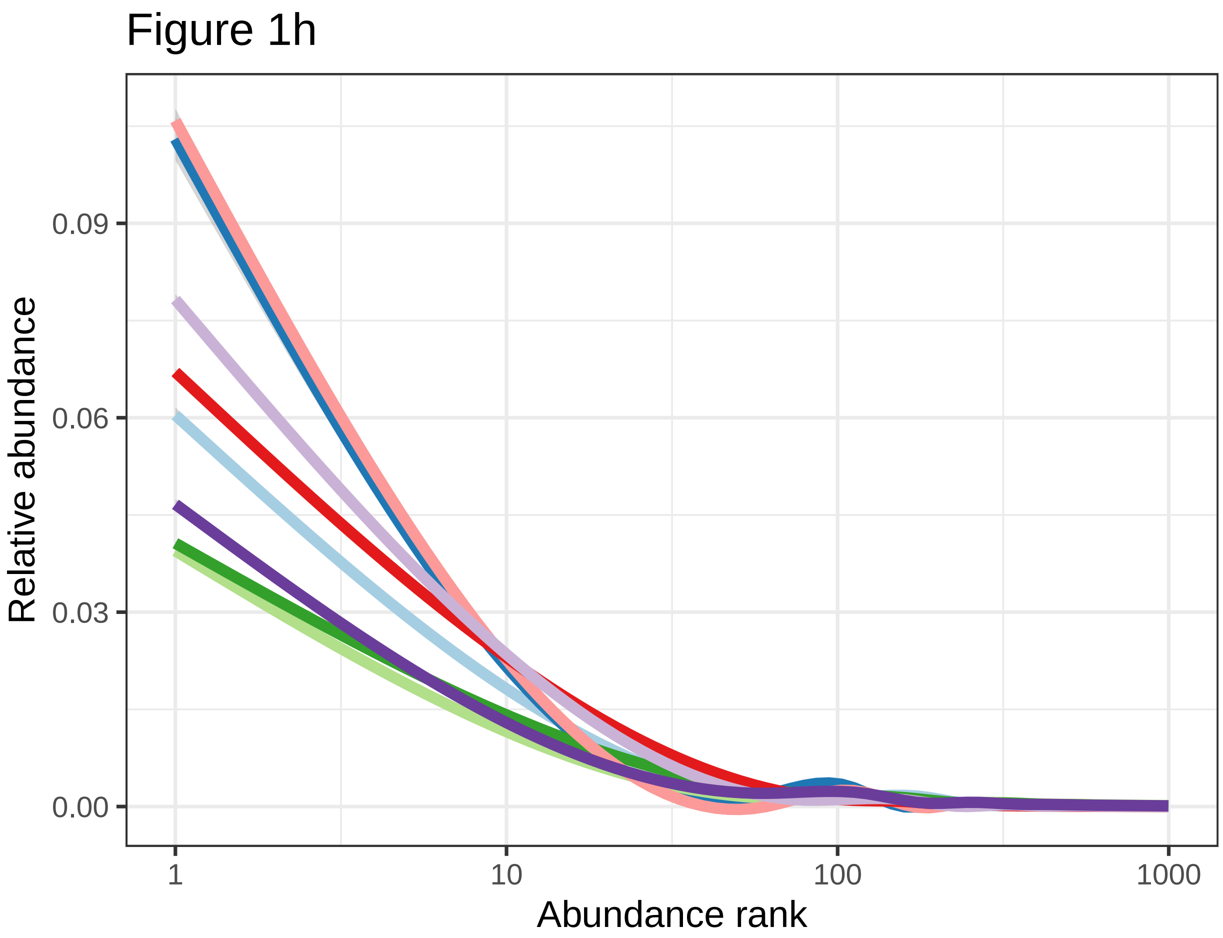


```
Fig1h<-ggplot(occupancy_abundance_df, aes(y = RelAbundance, x = Rank,
  group = host_species,  col = host_species))+
  geom_smooth(size = 2)+
  theme_bw(base_size = 14)+
  scale_x_log10(limits = c(1,1000))+
  scale_color_manual(values = palette)+
  ylab("Relative abundance")+
  xlab("Abundance rank")+
  theme(legend.position = "none")

Fig1_lower<-ggarrange(Fig1e, Fig1f, Fig1g,Fig1h, ncol = 4, widths = c(1,1.3,1.2,1.2),
  labels = c("E", "F", "G", "H"))
```

```
#ggarrange(Fig1_upper, Fig1_lower, ncol=1, heights = c(1.1,1))
```

Figure 1 final

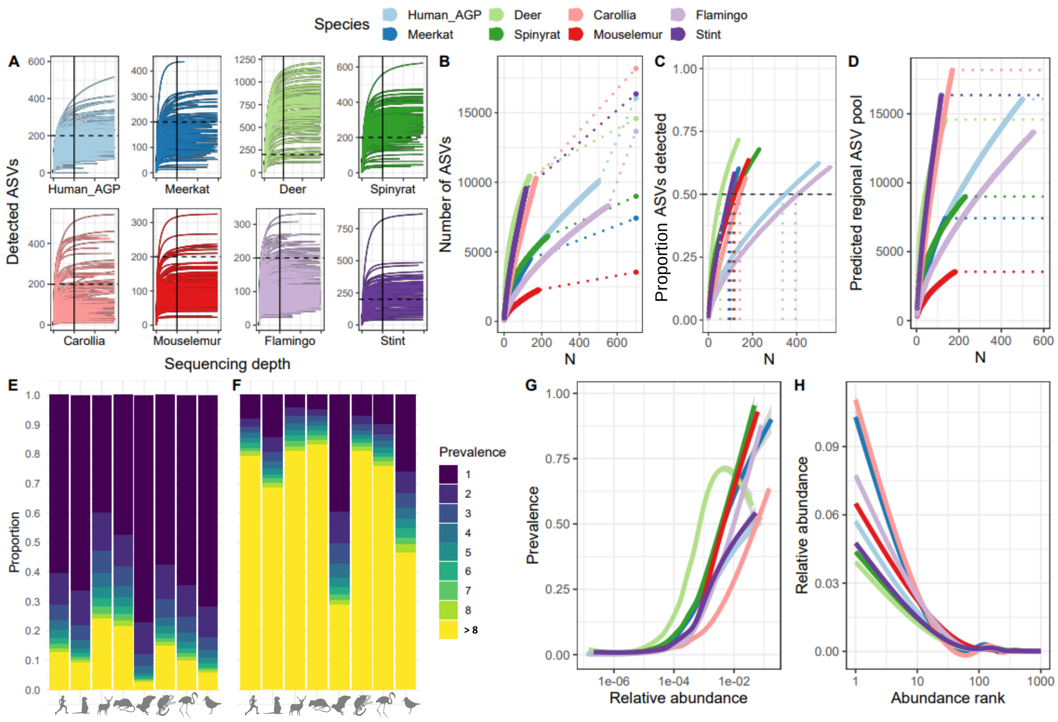

Figure 1: Figure 1

Calculate alpha diversity per sample

- Next we generate alpha diversity estimates for the four alpha

```
list1<-list()

uniq<-names(phylo_list)
thresholds<-c(0,0.1,0.2,0.3,0.4,0.5,0.6,0.7,0.8,0.9)

# for loop 1 to repeat for every species

for (j in 1:length(uniq)){

data<-phylo_list[[j]]  #for loop 1 (uniq)
#data<-phylo_list$Meerkat#for loop 1 (uniq)
data<-prune_taxa(taxa_sums(data) > 0, data)
data<-rarefy_even_depth(data, sample.size = 10000, rngsee = 100, replace = TRUE,
  trimOTUs=TRUE,verbose=FALSE)
```

```r
#### for loop 2 to repeat for every prevalence threshold
list2<-list()

## for loop 2 (thresholds)
for (i in 1:length(thresholds)){

   tryCatch({ #catch errors

data_subset<-microbiome::core(data, detection = 0, prevalence = thresholds[i]) #edit
alpha<-estimate_richness(data_subset, measures=c("Observed", "Shannon"))
alpha$Faiths<-metagMisc::phyloseq_phylo_div(data_subset, measures = c("PD"))$PD
alpha$BWPD<-estimate_bwpd(data_subset)$PSEs
alpha$Sample<-sample_names(data_subset)
alpha$Species<-as.character(uniq[[j]])
alpha$Prevalence<-thresholds[i] #edit
alpha$SampleSums<- sample_sums(data_subset)

list2[[i]]<-alpha

   }, error=function(e){})

}

alpha_df<-do.call(rbind, list2)

list1[[j]]<-alpha_df

}
```

```r
## combine
alpha_df_all<-do.call(rbind, list1)

### change NAs into zeros

alpha_df_all$Faiths[is.na(alpha_df_all$Faiths)] <- 0
alpha_df_all$BWPD[is.na(alpha_df_all$BWPD)] <- 0

################# generate standarised values per species

alpha_df_all<-transform(alpha_df_all, Observed_scaled=ave(Observed, Species, FUN=scale))
alpha_df_all<-transform(alpha_df_all, Faiths_scaled=ave(Faiths, Species, FUN=scale))
alpha_df_all<-transform(alpha_df_all, Shannon_scaled=ave(Shannon, Species, FUN=scale))
alpha_df_all<-transform(alpha_df_all, BWPD_scaled=ave(BWPD, Species, FUN=scale))
str(alpha_df_all)


alpha_df_all<-alpha_df_all[,c(5,6,7, 1,2,3,4,9,10,11, 12, 8)]

write.csv(alpha_df_all, "alpha_df_all_final.csv")
#alpha_df_all<-read.csv("alpha_df_all_final.csv", row.names = 1)
```

Calculate beta dissimilarity per sample

```
list1<-list()

uniq<-names(phylo_list)
thresholds<-c(0,0.1,0.2,0.3,0.4,0.5,0.6,0.7,0.8,0.9)

# for loop 1 to repeat for every species

for (j in 1:length(uniq)){

  data<-phylo_list[[j]]  #for loop 1 (uniq)
  data<-prune_taxa(taxa_sums(data) > 0, data)
  data<-rarefy_even_depth(data, sample.size = 10000, rngsee = 100, replace = TRUE,
    trimOTUs=TRUE,verbose=TRUE)


  #### for loop 2 to repeat for every prevalence threshold

  list2<-list()

  ## for loop 2 (thresholds)

  for (i in 1:length(thresholds)){

    tryCatch({ #catch errors

      #data_subset<-phyloseq_filter_prevalence(data, prev.trh = thresholds[i]) #edit
      data_subset<-microbiome::core(data, detection = 0, prevalence = thresholds[i]) #edit

      unifrac<-as.matrix(phyloseq::distance(data_subset, method = "unifrac"))
      wunifrac<-as.matrix(phyloseq::distance(data_subset, method = "wunifrac"))
      morisita<-as.matrix(phyloseq::distance(data_subset, method = "morisita"))
      jaccard<-as.matrix(phyloseq::distance(data_subset, method = "jaccard"))

      beta<-data.frame(colMeans(unifrac, na.rm=T))
      names(beta)<-"unifrac"
      beta$wunifrac<-colMeans(wunifrac, na.rm=T)
      beta$morisita<-colMeans(morisita, na.rm=T)
      beta$jaccard<-colMeans(jaccard, na.rm=T)

      beta$Sample<-sample_names(data_subset)
      beta$Species<-as.character(uniq[[j]])
      beta$Prevalence<-thresholds[i] #edit
      beta$SampleSums<-sample_sums(data_subset)

      list2[[i]]<-beta

    }, error=function(e){})

  }

  beta_df<-do.call(rbind, list2)
```

```
  list1[[j]]<-beta_df

}


beta_df_all<-do.call(rbind, list1)
write.csv(beta_df_all, "beta_df_all_final.csv")

#beta_df_all<-read.csv("beta_df_all_final.csv", row.names = 1)
```

Figure 2a-d: Relationship between alpha diversity and prevalence threshold

```
alpha_df_all<-read.csv("alpha_df_all_final.csv", row.names = 1)
beta_df_all<-read.csv("beta_df_all_final.csv", row.names = 1)

#alpha_df_all <- read.csv("C:/Users/risel/Dropbox/Sommer postdoc/Core microbiome project/NEW SUBMISSION

############# alpha diversity

alpha_df_all$Species<-factor(alpha_df_all$Species, levels = uniq)
alpha_df_all$Prevalence<-factor(alpha_df_all$Prevalence)

alpha_short_scaled<-alpha_df_all[,c(8,9,10,11,1,2,3)]

alpha_long_scaled<-gather(alpha_short_scaled, Index, Distance, Observed_scaled,
  Faiths_scaled, Shannon_scaled, BWPD_scaled, factor_key = TRUE)
alpha_long_scaled$Index<-factor(alpha_long_scaled$Index, level = c("Observed_scaled",
  "Faiths_scaled", "Shannon_scaled", "BWPD_scaled"))

alpha_short_unscaled<-alpha_df_all[,c(4,5,6,7,1,2,3)]

alpha_long_unscaled<-gather(alpha_short_unscaled, Index, Distance, Observed, Faiths,
  Shannon, BWPD, factor_key = TRUE)
alpha_long_unscaled$Index<-factor(alpha_long_unscaled$Index, level = c("Observed",
  "Faiths", "Shannon", "BWPD"))


##############################

ggplot(alpha_long_scaled, aes(x =Prevalence, y = Distance))+
  geom_jitter(aes(fill = Species, col = Species), pch=21, size=0.5,  alpha = 0.7,
    width =0.3)+
  facet_wrap(~Species+Index, ncol = 4, scales = "free_y")+
  scale_fill_manual(values = palette)+
  scale_color_manual(values = palette)+
  theme(axis.text.x = element_text(angle = 90, hjust = 1))+
  theme_bw(base_size = 14)+
  ylab("")+
  theme(panel.grid.major = element_blank(), panel.grid.minor = element_blank())+
  theme(axis.title.x=element_blank(),axis.text.x=element_blank(),
    axis.ticks.x=element_blank())+   theme(panel.grid.major = element_blank(),
```

```
      panel.grid.minor = element_blank())+
theme(legend.position="none")+
# theme(strip.background = element_blank(), strip.text.x = element_blank())+
 ggtitle("Figure 2a-d: Scaled alpha diversity")
```

Figure 2a−d: Scaled alpha diversity

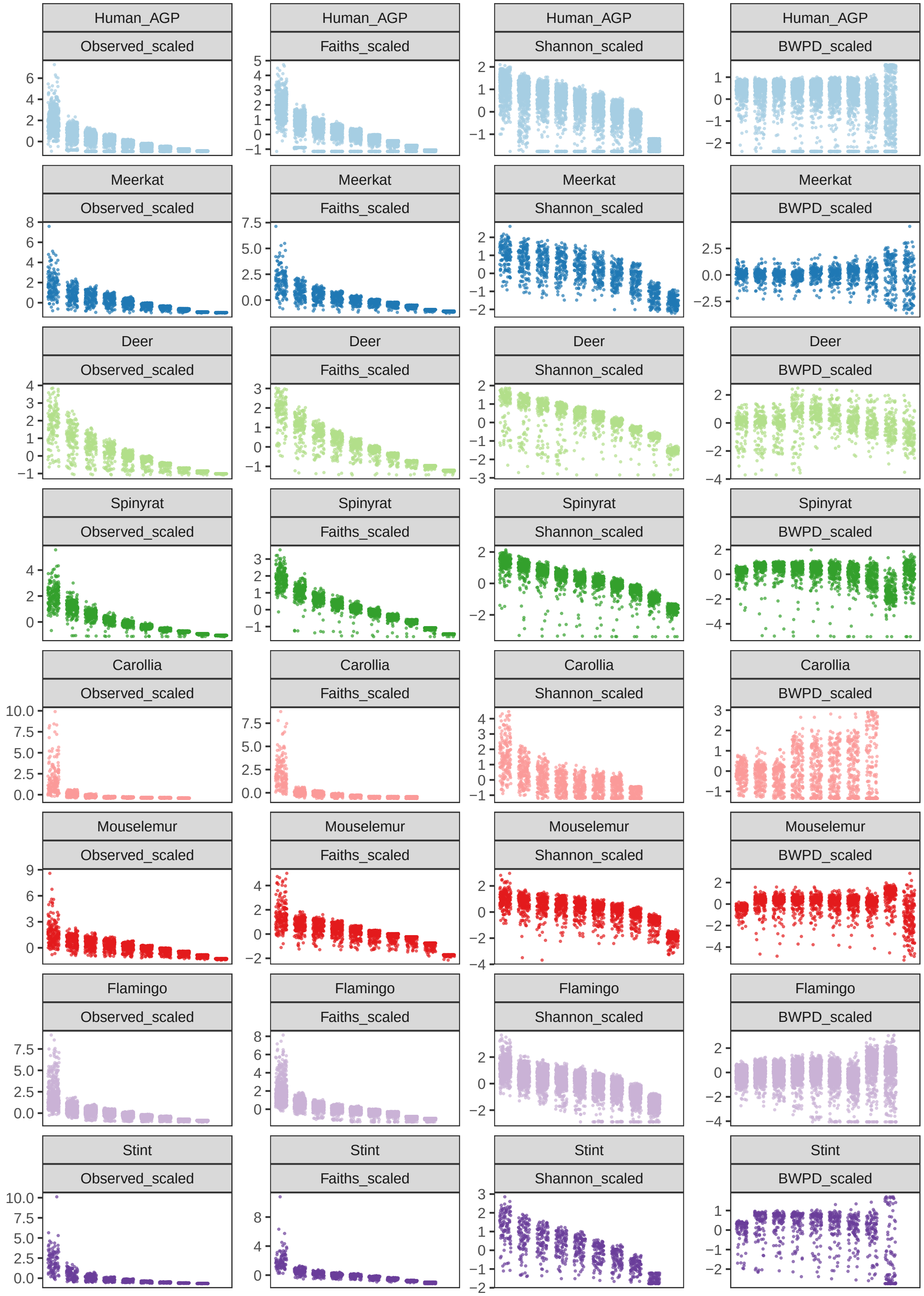

```r
alpha_plot<-ggplot(alpha_long_scaled, aes(x =Prevalence, y = Distance))+
  geom_jitter(aes(fill = Species, col = Species), pch=21, size=0.5,  alpha = 0.7,
    width =0.3)+
  facet_wrap(~Species+Index, ncol = 4, scales = "free_y")+
  scale_fill_manual(values = palette)+
  scale_color_manual(values = palette)+
  theme(axis.text.x = element_text(angle = 90, hjust = 1))+
  theme_bw(base_size = 14)+
  ylab("")+
  theme(panel.grid.major = element_blank(), panel.grid.minor = element_blank())+
  theme(axis.title.x=element_blank(),axis.text.x=element_blank(),
    axis.ticks.x=element_blank())+   theme(panel.grid.major = element_blank(),
      panel.grid.minor = element_blank())+
  theme(legend.position="none")+
  theme(strip.background = element_blank(), strip.text.x = element_blank())

## unscaled alpha diversity

ggplot(alpha_long_unscaled, aes(x =Prevalence, y = Distance))+
  geom_jitter(aes(fill = Species, col = Species), pch=21, size=0.5,  alpha = 0.7,
    width =0.3)+
  facet_wrap(~Species+Index, ncol = 4, scales = "free_y")+
  scale_fill_manual(values = palette)+
  scale_color_manual(values = palette)+
  theme(axis.text.x = element_text(angle = 90, hjust = 1))+
  theme_bw(base_size = 14)+
  ylab("")+
  theme(panel.grid.major = element_blank(), panel.grid.minor = element_blank())+
  theme(axis.title.x=element_blank(),axis.text.x=element_blank(),
    axis.ticks.x=element_blank())+
  theme(panel.grid.major = element_blank(), panel.grid.minor = element_blank())+
  theme(legend.position="none")+
 # theme(strip.background = element_blank(), strip.text.x = element_blank())+
  ggtitle("Sup. figure: Unscaled alpha diversity")
```

Sup. figure: Unscaled alpha diversity

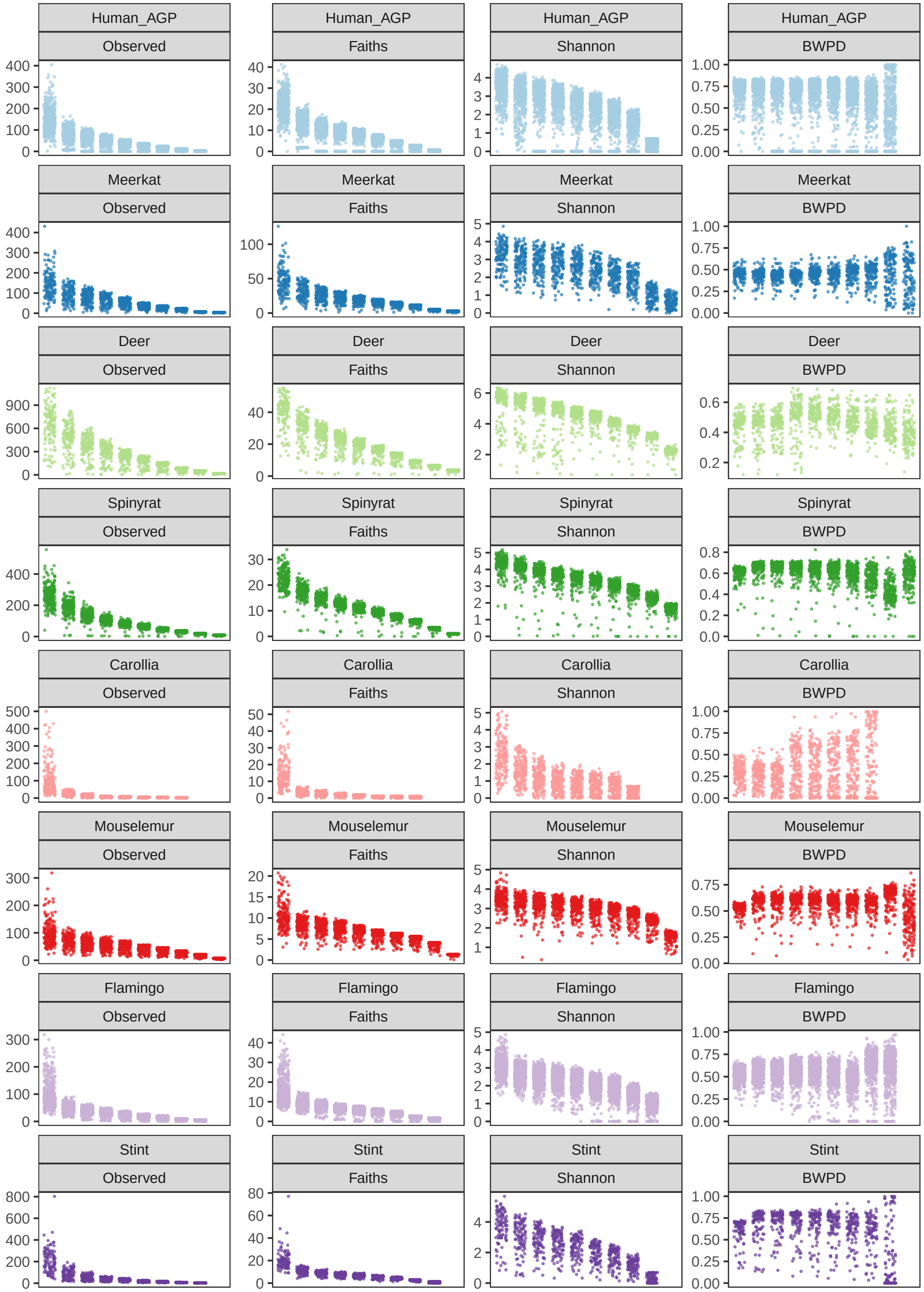

Figure 2e-h: Relationship between beta dissimilarity and prevalence threshold

```
#beta_df_all <- read.csv("C:/Users/risel/Dropbox/Sommer postdoc/Core microbiome project/NEW SUBMISSION/

beta_df_all$Species<-factor(beta_df_all$Species, levels = uniq)
beta_df_all$Prevalence<-factor(beta_df_all$Prevalence)
beta_short_normal<-beta_df_all[,c(1:7)]
beta_long_normal<-gather(beta_short_normal, Index, Distance, jaccard, unifrac,
  morisita,  wunifrac, factor_key = TRUE)
beta_long_normal$Index<-factor(beta_long_normal$Index, level = c("jaccard",
  "unifrac",  "morisita",  "wunifrac"))

ggplot(beta_long_normal, aes(x =Prevalence, y = Distance))+
  geom_jitter(aes(fill = Species, col = Species), pch=21, size=0.2, alpha = 0.7,
    width =0.3)+
  facet_wrap(~Species+Index, ncol = 4, scales = "free_y")+
  scale_fill_manual(values = palette)+
  scale_color_manual(values = palette)+
  theme(axis.text.x = element_text(angle = 90, hjust = 1))+
  theme_bw(base_size = 14)+
  ylab("")+
  ylim(0,1)+
  theme(panel.grid.major = element_blank(), panel.grid.minor = element_blank())+
  theme(axis.title.x=element_blank(),axis.text.x=element_blank(),
    axis.ticks.x=element_blank())+
  #theme(panel.grid.major = element_blank(), panel.grid.minor = element_blank())+
  theme(legend.position="none")+
  ggtitle("Figure 2e-h: beta dissimilarity")
```

Figure 2e−h: beta dissimilarity

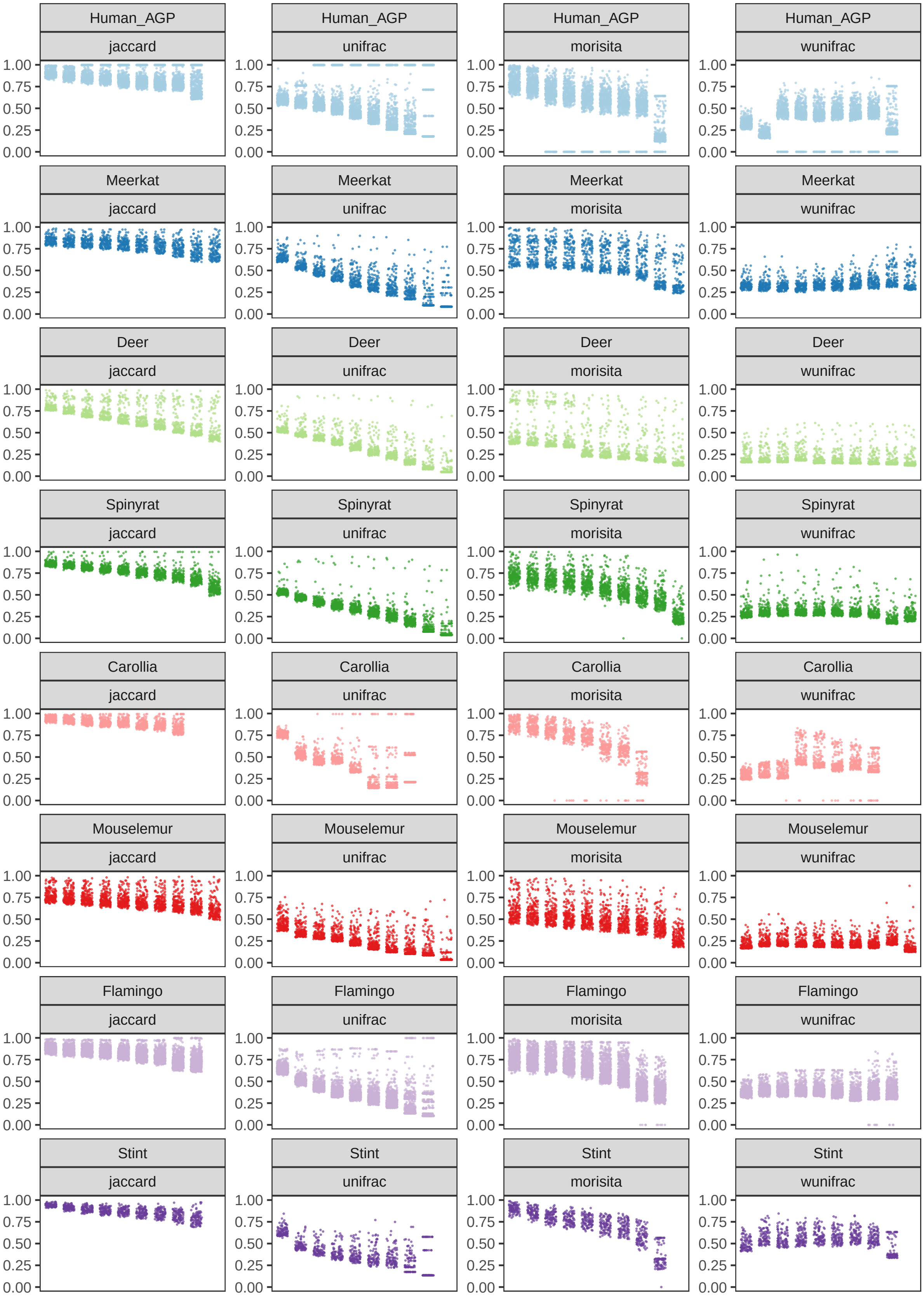

```
beta_plot<-ggplot(beta_long_normal, aes(x =Prevalence, y = Distance))+
  geom_jitter(aes(fill = Species, col = Species), pch=21, size=0.2, alpha = 0.7,
    width =0.3)+
 # geom_boxplot(alpha = 0.7, outlier.shape = NA) +
  facet_wrap(~Species+Index, ncol = 4, scales = "free_y")+
  scale_fill_manual(values = palette)+
  scale_color_manual(values = palette)+
  theme(axis.text.x = element_text(angle = 90, hjust = 1))+
  theme_bw(base_size = 14)+
  ylab("")+
  ylim(0,1)+
  theme(panel.grid.major = element_blank(), panel.grid.minor = element_blank())+
  theme(axis.title.x=element_blank(),axis.text.x=element_blank(),
    axis.ticks.x=element_blank())+
  theme(panel.grid.major = element_blank(), panel.grid.minor = element_blank())+
  theme(legend.position="none")+
  theme(strip.background = element_blank(), strip.text.x = element_blank())
```

Bartlett's Test for changes in variance

```
bartlett.test(alpha_short_scaled$Observed_scaled, alpha_short_scaled$Prevalence)
```

```
##
##  Bartlett test of homogeneity of variances
##
## data:  alpha_short_scaled$Observed_scaled and alpha_short_scaled$Prevalence
## Bartlett's K-squared = 23107, df = 9, p-value < 2.2e-16
```

```
bartlett.test(alpha_short_scaled$Faiths_scaled, alpha_short_scaled$Prevalence)
```

```
##
##  Bartlett test of homogeneity of variances
##
## data:  alpha_short_scaled$Faiths_scaled and alpha_short_scaled$Prevalence
## Bartlett's K-squared = 14281, df = 9, p-value < 2.2e-16
```

```
bartlett.test(alpha_short_scaled$Shannon_scaled, alpha_short_scaled$Prevalence)
```

```
##
##  Bartlett test of homogeneity of variances
##
## data:  alpha_short_scaled$Shannon_scaled and alpha_short_scaled$Prevalence
## Bartlett's K-squared = 1166.4, df = 9, p-value < 2.2e-16
```

```
bartlett.test(alpha_short_scaled$BWPD_scaled, alpha_short_scaled$Prevalence)
```

```
##
##  Bartlett test of homogeneity of variances
##
## data:  alpha_short_scaled$BWPD_scaled and alpha_short_scaled$Prevalence
## Bartlett's K-squared = 2377.1, df = 9, p-value < 2.2e-16
```

```
bartlett.test(beta_df_all$jaccard, beta_df_all$Prevalence)
```

```
##
##  Bartlett test of homogeneity of variances
##
## data:  beta_df_all$jaccard and beta_df_all$Prevalence
## Bartlett's K-squared = 1619.8, df = 9, p-value < 2.2e-16
```

```
bartlett.test(beta_df_all$unifrac, beta_df_all$Prevalence)
```

```
##
##  Bartlett test of homogeneity of variances
##
## data:  beta_df_all$unifrac and beta_df_all$Prevalence
## Bartlett's K-squared = 2499.8, df = 9, p-value < 2.2e-16
```

```
bartlett.test(beta_df_all$morisita, beta_df_all$Prevalence)
```

```
##
##  Bartlett test of homogeneity of variances
##
## data:  beta_df_all$morisita and beta_df_all$Prevalence
## Bartlett's K-squared = 316.55, df = 9, p-value < 2.2e-16
```

```
bartlett.test(beta_df_all$wunifrac, beta_df_all$Prevalence)
```

```
##
##  Bartlett test of homogeneity of variances
##
## data:  beta_df_all$wunifrac and beta_df_all$Prevalence
## Bartlett's K-squared = 743.65, df = 9, p-value < 2.2e-16
```

Figure 3: Mean changes to alpha and beta diversity with prevalence threshold

```
mean_alpha<-ddply(alpha_long_scaled, .(Species,Prevalence,Index), summarize,
  mean= mean(Distance))

Fig3a<-ggplot(alpha_long_scaled, aes(x =Prevalence, y = Distance, group = Species))+
  geom_smooth(aes(col = Species), alpha = 0.2, method = "loess", size = 1.5)+
  scale_color_manual(values = palette)+
  facet_wrap(~Index, ncol = 9)+
  theme_bw(base_size = 14)+
  theme( panel.grid.minor = element_blank())+
  theme(legend.position="none")+
 # theme(strip.background = element_blank(),strip.text.x = element_blank())+
  #geom_vline(xintercept = "0.7", linetype = "dashed")+
  theme(axis.title = element_blank())+
  theme(axis.text.x = element_text(angle = 45))+
  theme(plot.margin=unit(c(0.2,0.2,0.8,0.2),"cm"))+
```

```
  scale_x_discrete(breaks=c(0,0.2,0.4,0.6,0.8))


###########################

mean_beta<-ddply(beta_long_normal, .(Species,Prevalence,Index), summarize,
  mean= mean(Distance))


Fig3b<-ggplot(beta_long_normal, aes(x =Prevalence, y = Distance, group = Species))+
  geom_smooth(aes(col = Species), alpha = 0.2, method = "loess", size = 1.5)+
  scale_color_manual(values = palette)+
  facet_wrap(~Index, ncol = 8)+
  theme_bw(base_size = 14)+
  theme( panel.grid.minor = element_blank())+
  theme(legend.position="none")+
  theme(axis.title = element_blank())+
 # theme(strip.background = element_blank(), strip.text.x = element_blank())+
 # geom_vline(xintercept = "0.7", linetype = "dashed")+
  theme(axis.text.x = element_text(angle = 45))+
  scale_x_discrete(breaks=c(0,0.2,0.4,0.6,0.8))

# figure 3

ggarrange(Fig3a, Fig3b, ncol = 1, align = "v",  common.legend = T, legend = "right",
  heights = c(1.1,1))
```

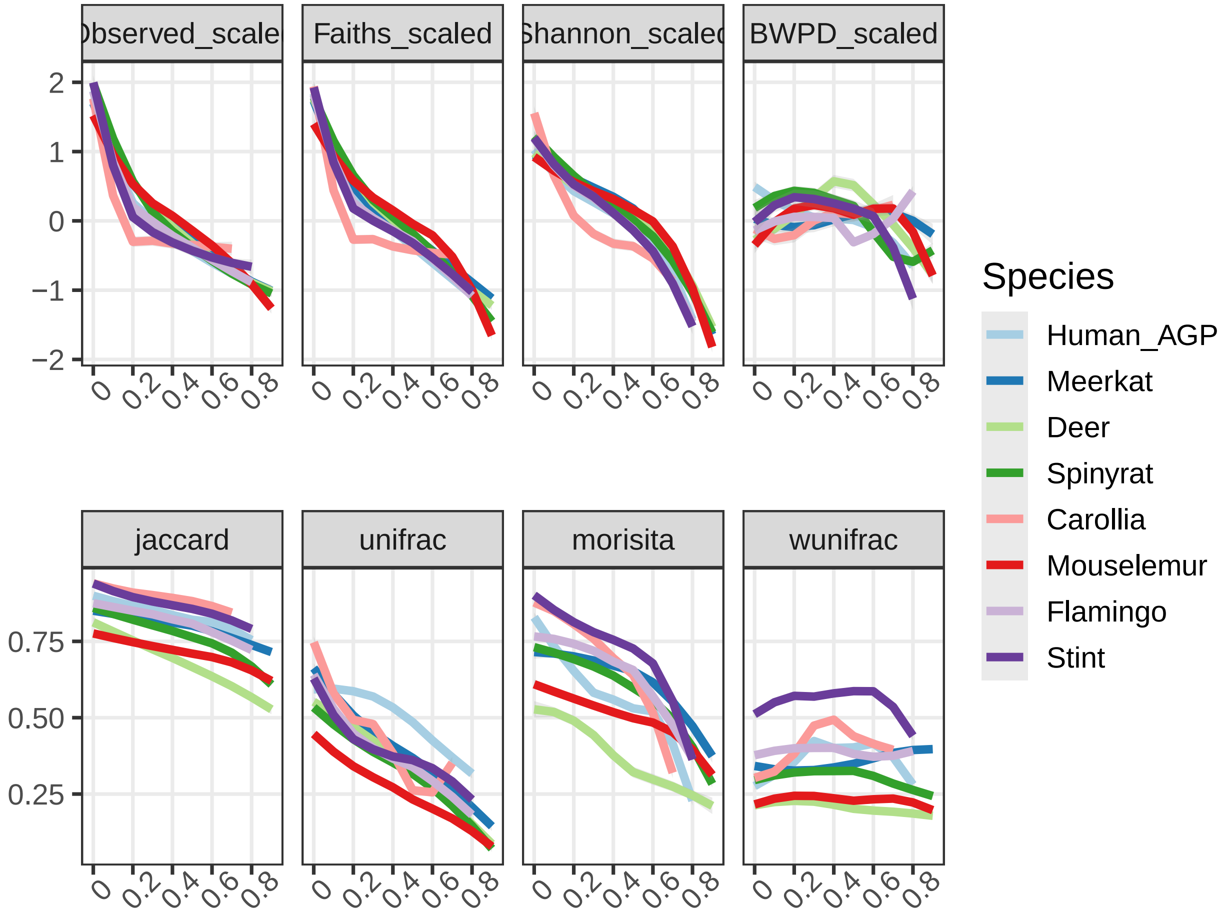

Figure S1: Correlation between sample read depth and diversity

```
seq_depth_corr<-alpha_df_all[,c("Observed_scaled","Faiths_scaled" , "Shannon_scaled", "BWPD_scaled","Sa

seq_depth_corr<- data.frame(seq_depth_corr, beta_df_all[,c("jaccard", "unifrac" , "morisita", "wunifrac


uniq_measures<-c("Observed_scaled","Faiths_scaled" , "Shannon_scaled", "BWPD_scaled", "jaccard", "unifra
uniq_species<-as.character(unique(alpha_df_all$Species))
uniq_prev<-c(0,0.1,0.2,0.3,0.4,0.5,0.6,0.7,0.8,0.9)

corr_list_i<-list() # i measure
corr_list_j<-list() # j threshold
corr_list_k<-list() # k species


for (i in 1:length(uniq_measures)){

 # data_1<-subset(correlations_df, Measure == uniq_measures[[i]])
 # data_1<-seq_depth_corr[,c("Observed_scaled","Sample","Species","Prevalence","SampleSums")]
  data_1<-seq_depth_corr[,c(uniq_measures[[i]],"Sample","Species","Prevalence","SampleSums")]
```

```
 for (k in 1:length(uniq_species)){

   data_2<-subset(data_1, Species == uniq_species[[k]])
   # data_2<-subset(data_1, Species == "Meerkat")

 for (j in 1:length(uniq_prev)){

   tryCatch({ #catch errors

    # data_3<-subset(data_2, Prevalence == "0.1")
     data_3<-subset(data_2, Prevalence == uniq_prev[[j]])

  test<-cor.test(data_3[,1], data_3[,5], method = "spearman", exact=FALSE)
  df<-data.frame(test$estimate)
  df$pval<-test$p.value
  names(df)[1]<-"rho"
  df$Prevelance<-uniq_prev[j]
  df$Species<-uniq_species[k]
  df$Measure<-uniq_measures[i]

 corr_list_j[[j]]<-df

   }, error=function(e){})

 }
   results_j<- do.call(rbind, corr_list_j)
   corr_list_k[[k]]<-results_j
 }

 results_k<- do.call(rbind, corr_list_k)
   corr_list_i[[i]]<-results_k
}

results_i<- do.call(rbind, corr_list_i)

results_i$Significant<-ifelse(results_i$pval < 0.05, "Sig", "Non_sig")
results_i$Prevelance<-as.numeric(results_i$Prevelance)
results_i$Measure<-factor(results_i$Measure, levels = c("Observed_scaled","Faiths_scaled" , "Shannon_sca
results_i<-subset(results_i, Prevelance != 0)

results_i$Measure<-revalue(results_i$Measure, c("Observed_scaled"="Observed", "Faiths_scaled" = "Faiths
  "jaccard" = "Jaccard", "unifrac" = "Unweighted Unifrac", "morisita" = "Morisita", "wunifrac" = "Weigh

results_i$Species<-factor(results_i$Species, levels = uniq)

ggplot(results_i, aes(x = Prevelance, y = rho))+
  stat_smooth(method = "loess", alpha = 0.7, col = "black", linetype=0, level = 0.95)+
  geom_point(aes(col = Species, shape = Significant), size = 2)+
  geom_line(aes(col = Species,  group = Species))+
  facet_wrap(~Measure, ncol = 4)+
  scale_color_manual(values = palette)+
  scale_shape_manual(values = c(4,15))+
 # geom_hline(yintercept = 0.6, linetype = "dashed")+
```

```
  geom_hline(yintercept = 0)+
  theme_bw(base_size = 14)+
  scale_x_continuous(breaks = c(0.1,0.2,0.3,0.4,0.5, 0.6,0.7,0.8,0.9))+
    scale_y_continuous(breaks = c(-0.8,-0.6, -0.4,-0.2, 0, 0.2, 0.4, 0.6, 0.8, 1.0))+
    theme(axis.text.x = element_text(angle = 45, hjust = 1))+
  ylab("Spearman's rho")+
  xlab("Prevelance threshold")
```

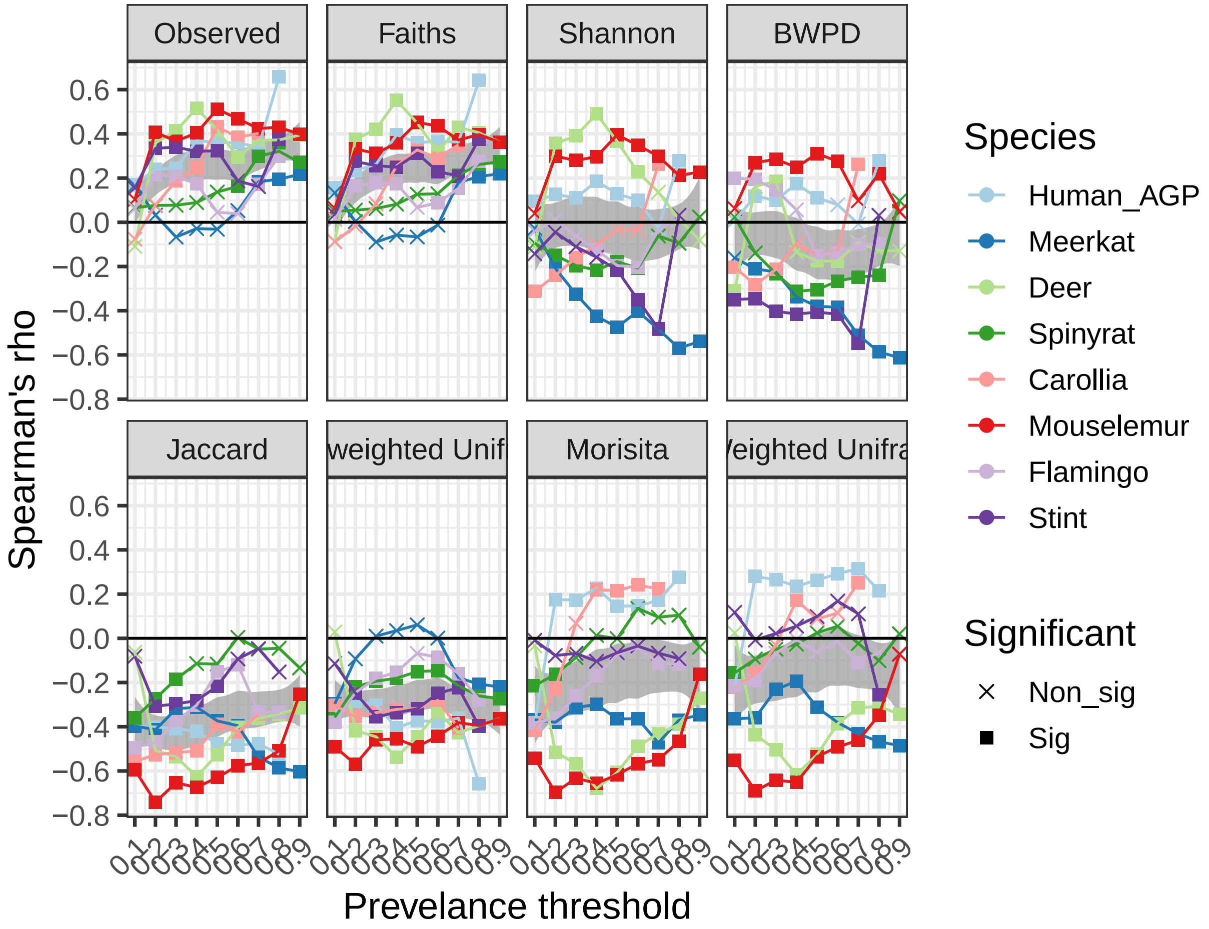

Visualise diversity correlation matrix with ggparirs

- Correlate sample level alpha and beta scores with original scores from unfiltered data.
- Probably a quicker way of doing this but I am sick of loops.
- Extra plots = ggally correlation matrix (with 0,0.2,0.4,0.6,0.8)

```
### observed

alpha_observed<-alpha_short_scaled[,c("Observed_scaled", "Sample", "Species",
  "Prevalence")]
alpha_corr_Observed <- spread(alpha_observed, Prevalence, Observed_scaled)

prev_levels<-levels(alpha_short_scaled$Prevalence)
```

```
prev_levels<-prev_levels[c(1,3,5,7,9)]

alpha_corr_Observed  %>%
  select(Species, where(is.numeric)) %>%
  GGally::ggpairs(mapping = aes(color = Species),
    columns = prev_levels,
    upper = list(continuous = wrap("cor", size = 3,  method = "spearman")),
   lower = list(continuous = wrap("smooth", alpha = 0.3, size=0.1))) +
  scale_colour_manual(values = palette) +
  scale_fill_manual(values = palette)+
   theme_bw(base_size = 14)+
  ggtitle("Spearman's correlations observed")
```

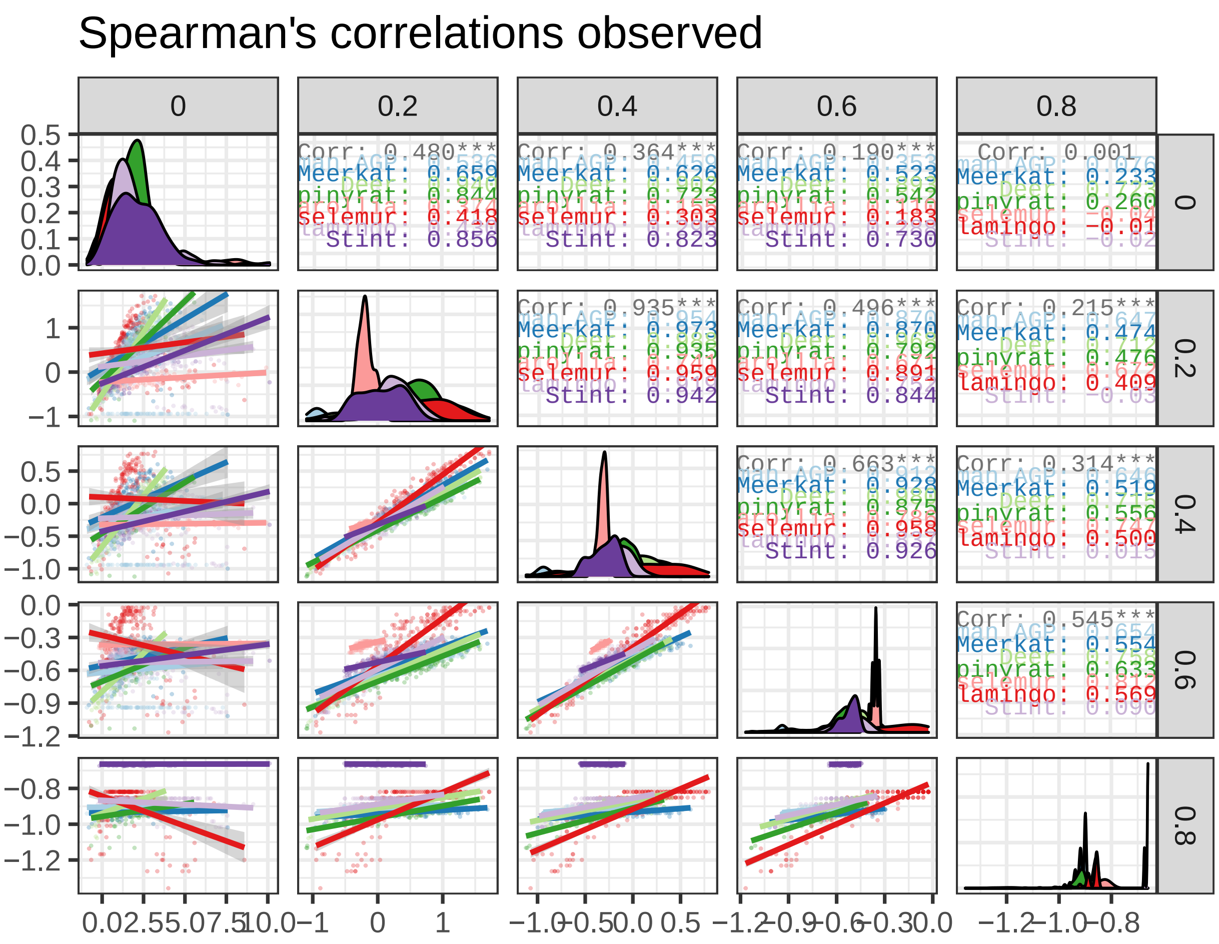


```
alpha_corr_Observed$Diversity<-"Alpha"
alpha_corr_Observed$Measure<-"Observed"

####### faiths

alpha_Faiths<-alpha_short_scaled[,c("Faiths_scaled", "Sample", "Species", "Prevalence")]
alpha_corr_Faiths <- spread(alpha_Faiths, Prevalence, Faiths_scaled)

alpha_corr_Faiths  %>%
```

```
  select(Species, where(is.numeric)) %>%
  GGally::ggpairs(mapping = aes(color = Species),
    columns = prev_levels,
    upper = list(continuous = wrap("cor", size = 3,  method = "spearman")),
   lower = list(continuous = wrap("smooth", alpha = 0.3, size=0.1))) +
  scale_colour_manual(values = palette) +
  scale_fill_manual(values = palette)+
   theme_bw(base_size = 14)+
  ggtitle("Spearman's correlations Faiths")
```

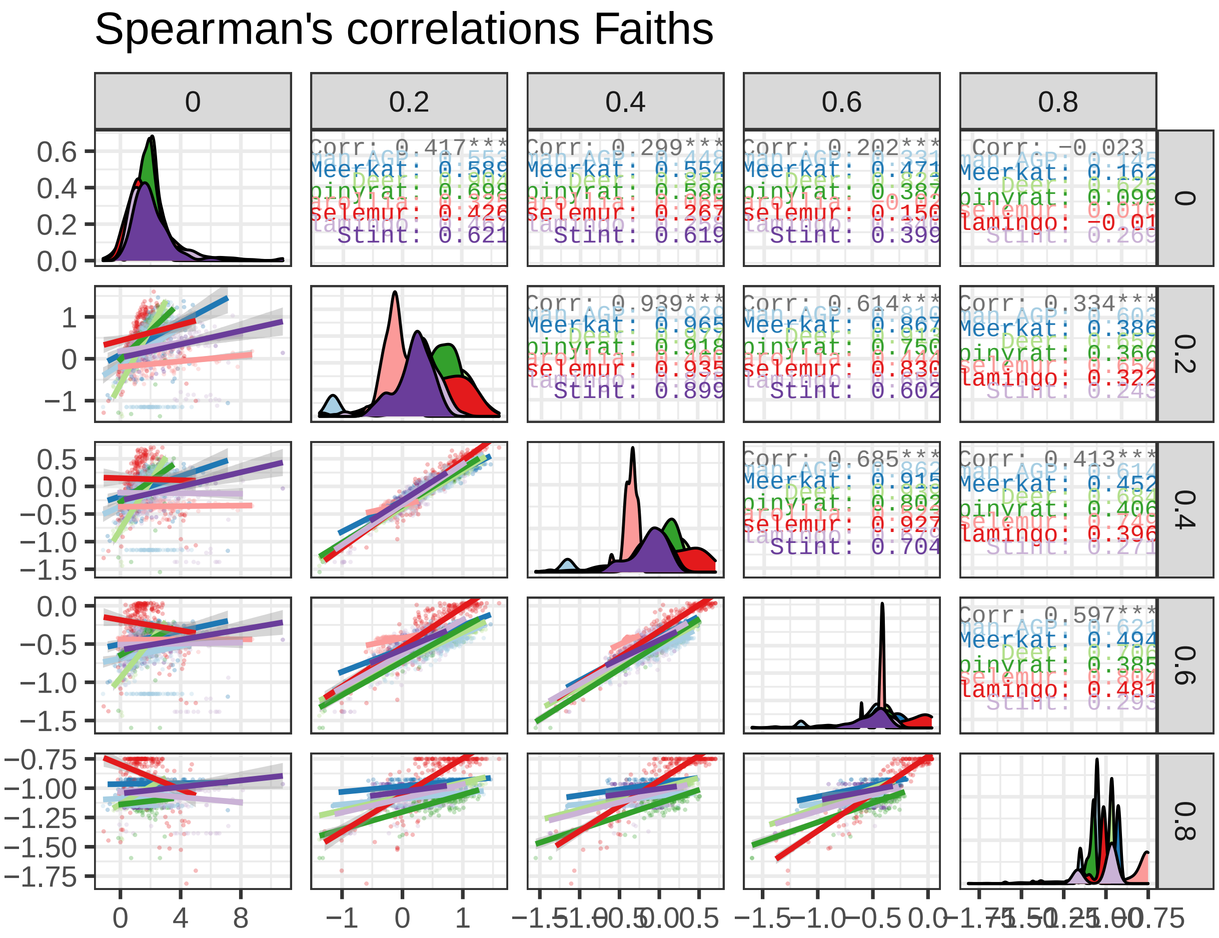


```
alpha_corr_Faiths$Diversity<-"Alpha"
alpha_corr_Faiths$Measure<-"Faiths"

##### shannon


alpha_shannon<-alpha_short_scaled[,c("Shannon_scaled", "Sample", "Species", "Prevalence")]
alpha_corr_Shannon <- spread(alpha_shannon, Prevalence, Shannon_scaled)

alpha_corr_Shannon  %>%
  select(Species, where(is.numeric)) %>%
  GGally::ggpairs(mapping = aes(color = Species),
```

```r
    columns = prev_levels,
    upper = list(continuous = wrap("cor", size = 3,  method = "spearman")),
   lower = list(continuous = wrap("smooth", alpha = 0.3, size=0.1))) +
  scale_colour_manual(values = palette) +
  scale_fill_manual(values = palette)+
   theme_bw(base_size = 14)+
  ggtitle("Spearman's correlations Shannon")
```

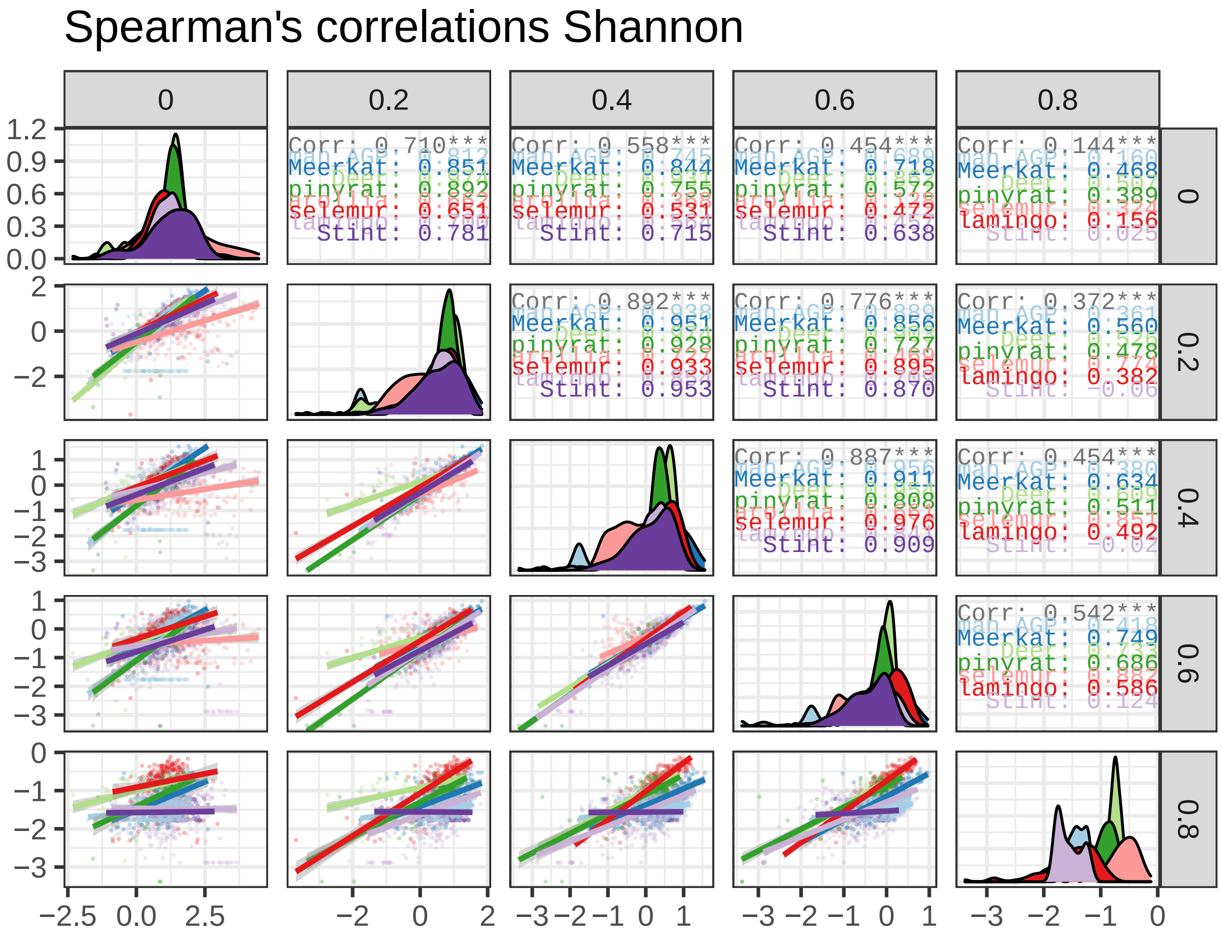


```r
alpha_corr_Shannon$Diversity<-"Alpha"
alpha_corr_Shannon$Measure<-"Shannon"


######## BWPD

alpha_BWPD<-alpha_short_scaled[,c("BWPD_scaled", "Sample", "Species", "Prevalence")]
alpha_corr_BWPD <- spread(alpha_BWPD, Prevalence, BWPD_scaled)


alpha_corr_BWPD  %>%
  select(Species, where(is.numeric)) %>%
  GGally::ggpairs(mapping = aes(color = Species),
    columns = prev_levels,
```

```
    upper = list(continuous = wrap("cor", size = 3,  method = "spearman")),
   lower = list(continuous = wrap("smooth", alpha = 0.3, size=0.1))) +
  scale_colour_manual(values = palette) +
  scale_fill_manual(values = palette)+
   theme_bw(base_size = 14)+
  ggtitle("Spearman's correlations BWPD")
```

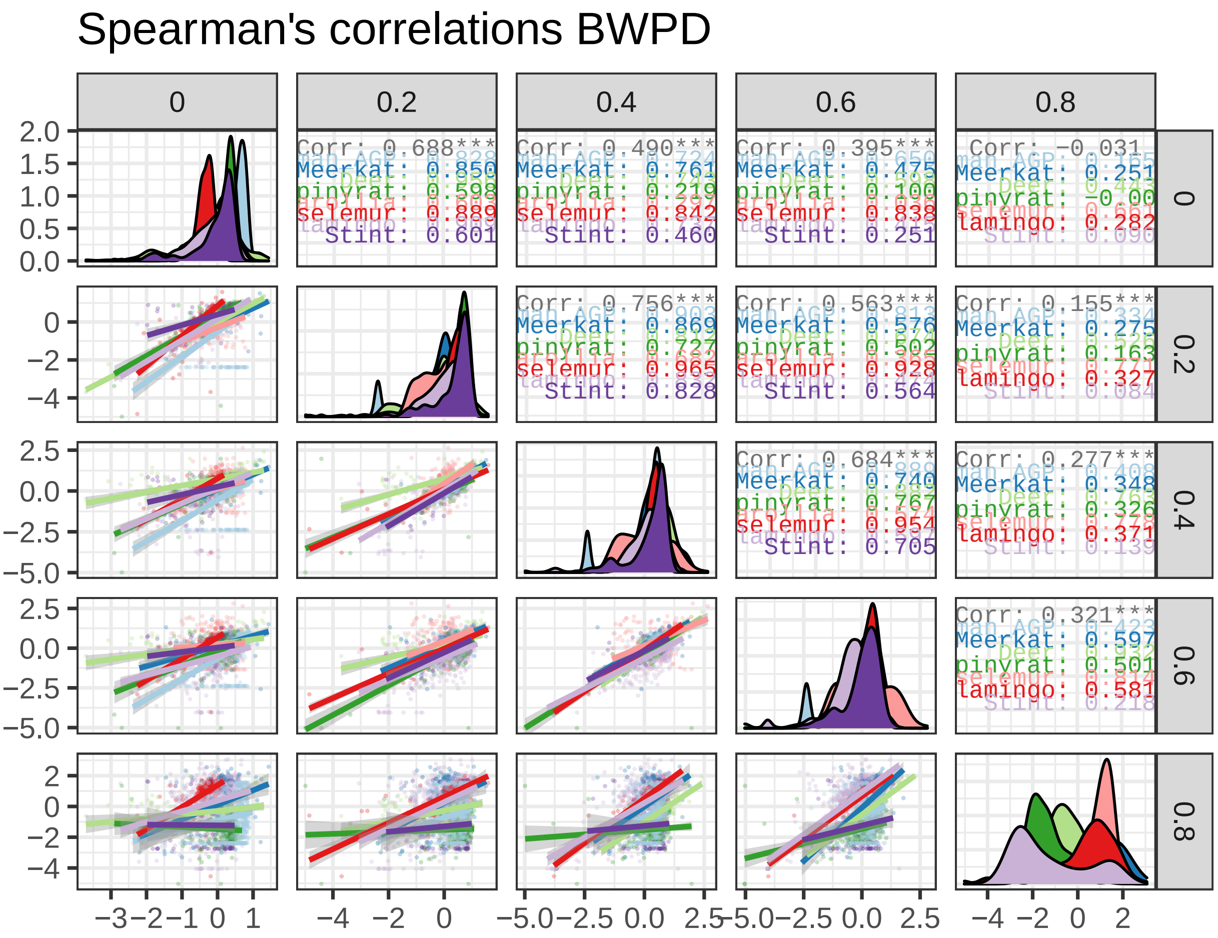


```
alpha_corr_BWPD$Diversity<-"Alpha"
alpha_corr_BWPD$Measure<-"BWPD"
```

```
### jaccard

beta_jaccard<-beta_short_normal[,c("jaccard", "Sample", "Species", "Prevalence")]
beta_corr_jaccard <- spread(beta_jaccard, Prevalence, jaccard)

beta_corr_jaccard %>%
  select(Species, where(is.numeric)) %>%
  GGally::ggpairs(mapping = aes(color = Species),
    columns = prev_levels,
    upper = list(continuous = wrap("cor", size = 3,  method = "spearman")),
   lower = list(continuous = wrap("smooth", alpha = 0.3, size=0.1))) +
```

```
  scale_colour_manual(values = palette) +
  scale_fill_manual(values = palette)+
  ggtitle("Spearman's correlations Jaccard")
```

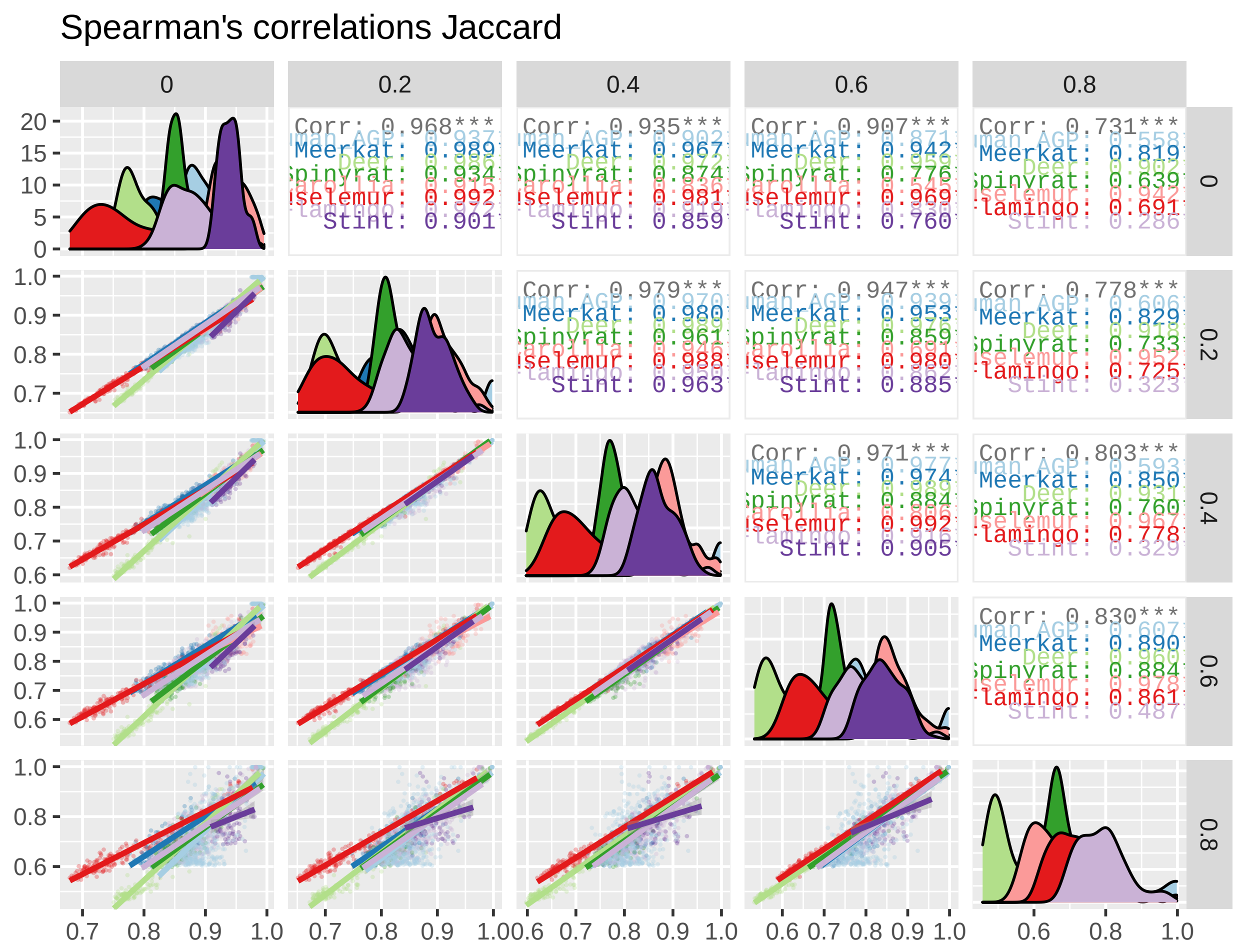


```
beta_corr_jaccard$Diversity<-"Beta"
beta_corr_jaccard$Measure<-"Jaccard"

### morisita

beta_morisita<-beta_short_normal[,c("morisita", "Sample", "Species", "Prevalence")]
beta_corr_morisita <- spread(beta_morisita, Prevalence, morisita)

beta_corr_morisita %>%
  select(Species, where(is.numeric)) %>%
  GGally::ggpairs(mapping = aes(color = Species),
    columns = prev_levels,
    upper = list(continuous = wrap("cor", size = 3,  method = "spearman")),
   lower = list(continuous = wrap("smooth", alpha = 0.3, size=0.1))) +
  scale_colour_manual(values = palette) +
  scale_fill_manual(values = palette)+
  ggtitle("Spearman's correlations Morisita")
```

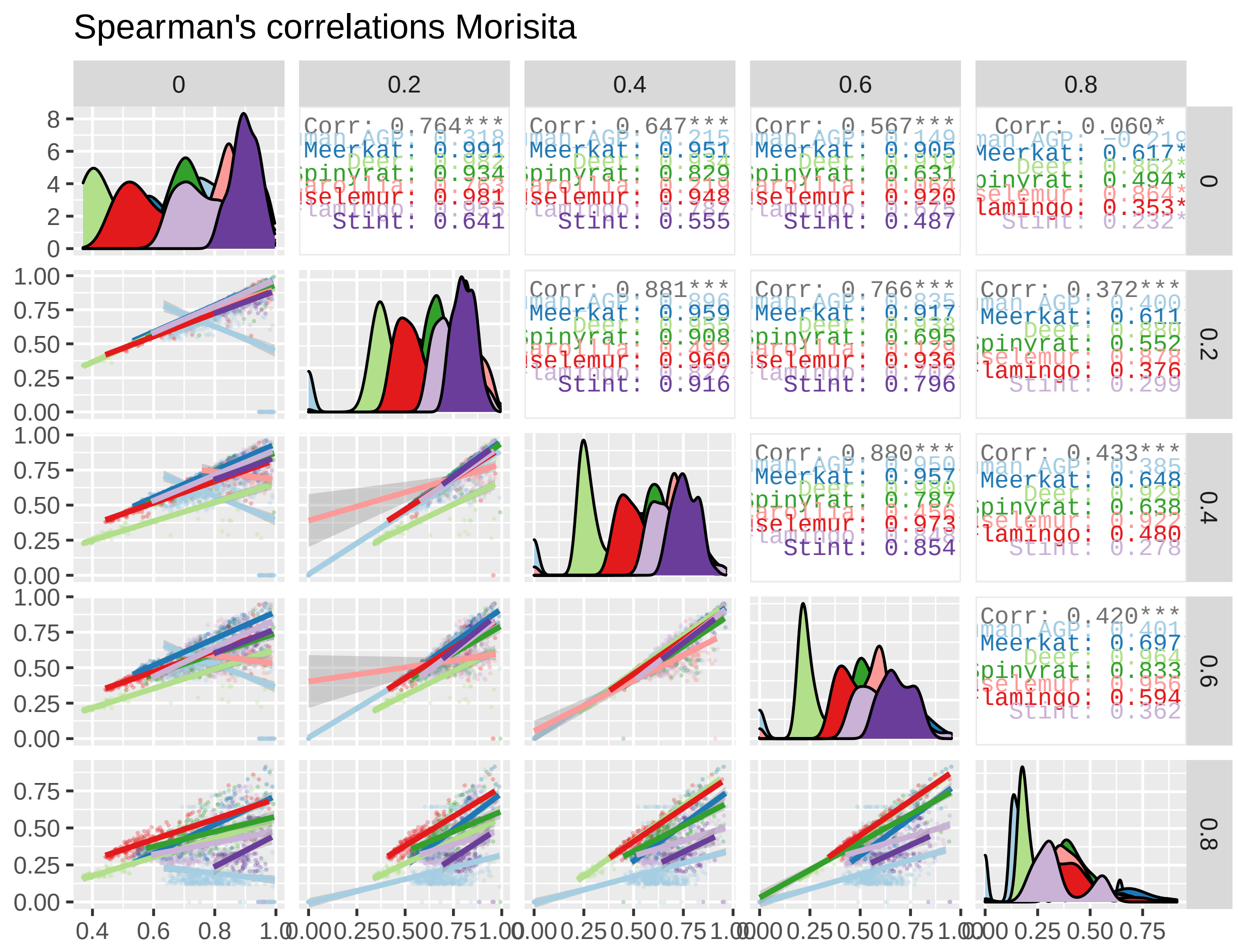


```
beta_corr_morisita$Diversity<-"Beta"
beta_corr_morisita$Measure<-"Morisita"


### unifrac

beta_unifrac<-beta_short_normal[,c("unifrac", "Sample", "Species", "Prevalence")]
beta_corr_unifrac <- spread(beta_unifrac, Prevalence, unifrac)

beta_corr_unifrac %>%
  select(Species, where(is.numeric)) %>%
  GGally::ggpairs(mapping = aes(color = Species),
    columns = prev_levels,
    upper = list(continuous = wrap("cor", size = 3,  method = "spearman")),
   lower = list(continuous = wrap("smooth", alpha = 0.3, size=0.1))) +
  scale_colour_manual(values = palette) +
  scale_fill_manual(values = palette)+
  ggtitle("Spearman's correlations Unifrac")
```

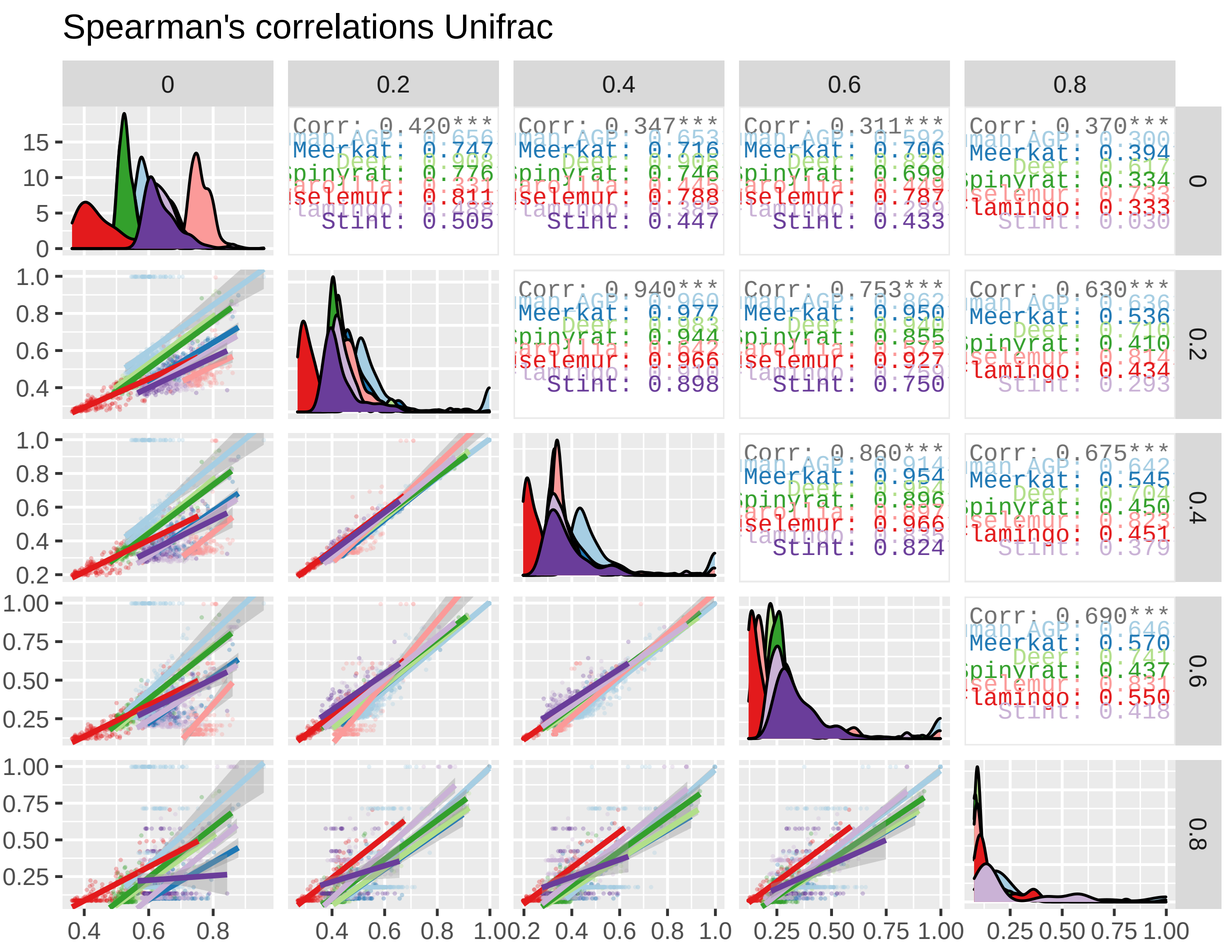


```
beta_corr_unifrac$Diversity<-"Beta"
beta_corr_unifrac$Measure<-"Unifrac"


### wunifrac

beta_wunifrac<-beta_short_normal[,c("wunifrac", "Sample", "Species", "Prevalence")]
beta_corr_wunifrac <- spread(beta_wunifrac, Prevalence, wunifrac)

beta_corr_wunifrac %>%
  select(Species, where(is.numeric)) %>%
  GGally::ggpairs(mapping = aes(color = Species),
    columns = prev_levels,
    upper = list(continuous = wrap("cor", size = 3,  method = "spearman")),
   lower = list(continuous = wrap("smooth", alpha = 0.3, size=0.1))) +
  scale_colour_manual(values = palette) +
  scale_fill_manual(values = palette)+
  ggtitle("Spearman's correlations Weighted Unifrac")
```

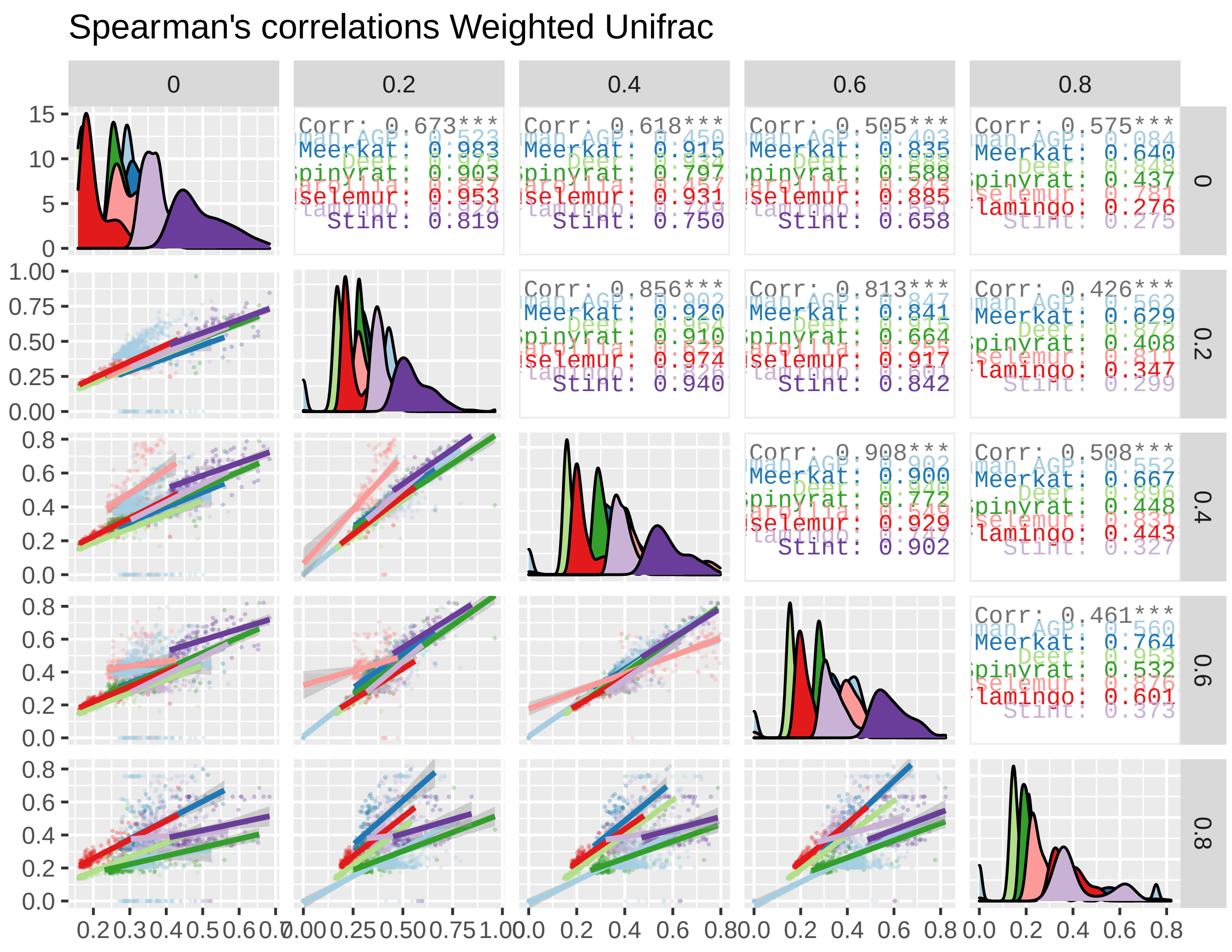


```r
beta_corr_wunifrac$Diversity<-"Beta"
beta_corr_wunifrac$Measure<-"Wunifrac"
```

Correlation between core diversity and unfiltered diversity

```r
# rbind dataframes together
correlations_df<-rbind(alpha_corr_Observed, alpha_corr_Faiths, alpha_corr_Shannon,
  alpha_corr_BWPD, beta_corr_jaccard, beta_corr_morisita, beta_corr_unifrac,
  beta_corr_wunifrac)

## change names of columns so they aren't numbers
names(correlations_df)[3]<-"Prev_0"
names(correlations_df)[4]<-"Prev_0.1"
names(correlations_df)[5]<-"Prev_0.2"
names(correlations_df)[6]<-"Prev_0.3"
names(correlations_df)[7]<-"Prev_0.4"
names(correlations_df)[8]<-"Prev_0.5"
names(correlations_df)[9]<-"Prev_0.6"
names(correlations_df)[10]<-"Prev_0.7"
names(correlations_df)[11]<-"Prev_0.8"
```

```r
names(correlations_df)[12]<-"Prev_0.9"

uniq_measures<- unique(correlations_df$Measure)
uniq_species<- unique(correlations_df$Species)
uniq_prevs<- c("Prev_0.1","Prev_0.2", "Prev_0.3", "Prev_0.4", "Prev_0.5", "Prev_0.6",
  "Prev_0.7", "Prev_0.8", "Prev_0.9")

## mega loop which somehow worked
# for each species, diversity measure, and threshold, correlate with original data

corr_list_i<-list() # i measure
corr_list_j<-list() # j threshold
corr_list_k<-list() # k species


for (i in 1:length(uniq_measures)){

  data_1<-subset(correlations_df, Measure == uniq_measures[[i]])
 # data_1<-subset(correlations_df, Measure == "Observed")

  for (k in 1:length(uniq_species)){

    data_2<-subset(data_1, Species == uniq_species[[k]])
    # data_2<-subset(data_1, Species == "Meerkat")

 for (j in 1:length(uniq_prevs)){

    tryCatch({ #catch errors

   test<-cor.test(data_2$Prev_0, data_2[,uniq_prevs[j]], method = "spearman", exact=FALSE)
   df<-data.frame(test$estimate)
   df$pval<-test$p.value
   names(df)[1]<-"rho"
   df$Prevelance<-uniq_prevs[j]
   df$Species<-uniq_species[k]
   df$Measure<-uniq_measures[i]

  corr_list_j[[j]]<-df

    }, error=function(e){})

 }
    results_j<- do.call(rbind, corr_list_j)
    corr_list_k[[k]]<-results_j
  }

  results_k<- do.call(rbind, corr_list_k)
    corr_list_i[[i]]<-results_k
}

results_i<- do.call(rbind, corr_list_i)
```

Figure 4: Spearman's correlation with unfiltered data

```
results_i$Measure<-factor(results_i$Measure, levels = c("Observed", "Faiths", "Shannon",
  "BWPD", "Jaccard", "Unifrac", "Morisita", "Wunifrac"))

results_i$Significant<-ifelse(results_i$pval < 0.05, "Sig", "Non_sig")

results_i$Prevelance<-revalue(results_i$Prevelance, c("Prev_0.1"=0.1, "Prev_0.2"=0.2,
  "Prev_0.3"=0.3, "Prev_0.4"=0.4, "Prev_0.5"=0.5, "Prev_0.6"=0.6, "Prev_0.7"=0.7,
  "Prev_0.8"=0.8, "Prev_0.9"=0.9))

results_i$Prevelance<-as.numeric(results_i$Prevelance)

ggplot(results_i, aes(x = Prevelance, y = rho))+
  stat_smooth(method = "loess", alpha = 0.7, col = "black", linetype=0, level = 0.95)+
  geom_point(aes(col = Species, shape = Significant), size = 2)+
  geom_line(aes(col = Species,  group = Species))+
  facet_wrap(~Measure, ncol = 4)+
  scale_color_manual(values = palette)+
  scale_shape_manual(values = c(15,16))+
  geom_hline(yintercept = 0.6, linetype = "dashed")+
  geom_hline(yintercept = 0)+
  theme_bw(base_size = 14)+
  scale_x_continuous(breaks = c(0.1,0.2,0.3,0.4,0.5, 0.6,0.7,0.8,0.9))+
    scale_y_continuous(breaks = c(-0.2, 0, 0.2, 0.4, 0.6, 0.8, 1.0))+
    theme(axis.text.x = element_text(angle = 45, hjust = 1))+
  ylab("Spearman's rho")+
  xlab("Prevelance threshold")+
  ggtitle("Figure 4")
```

Figure 4

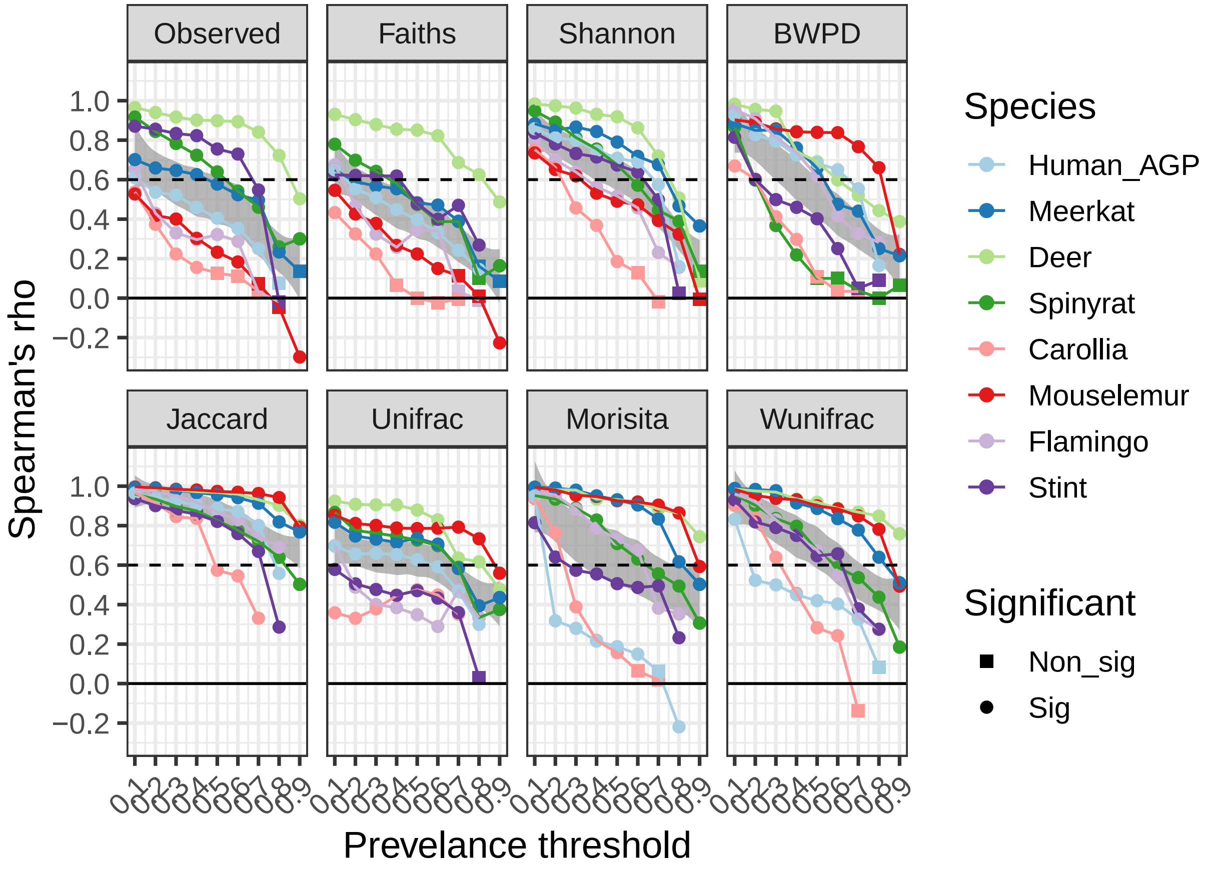


```
Fig4<-ggplot(results_i, aes(x = Prevelance, y = rho))+
  stat_smooth(method = "loess", alpha = 0.7, col = "black", linetype=0, level = 0.95)+
  geom_point(aes(col = Species, shape = Significant), size = 2)+
  geom_line(aes(col = Species,  group = Species))+
  facet_wrap(~Measure, ncol = 4)+
  scale_color_manual(values = palette)+
    scale_shape_manual(values = c(15,16))+
  geom_hline(yintercept = 0.6, linetype = "dashed")+
  geom_hline(yintercept = 0)+
  theme_bw(base_size = 14)+
  scale_x_continuous(breaks = c(0.1,0.2,0.3,0.4,0.5, 0.6,0.7,0.8,0.9))+
  scale_y_continuous(breaks = c(-0.2, 0, 0.2, 0.4, 0.6, 0.8, 1.0))+
    theme(axis.text.x = element_text(angle = 45, hjust = 1))+
  theme(strip.background = element_blank(), strip.text.x = element_blank())+
  ylab("Spearman's rho")+
  xlab("Prevelance threshold")+
  theme(panel.spacing.y = unit(3, "lines"))
```

Mean Spearman's correlation per measure and threshold

```r
results_i %>% group_by(Measure) %>%
  summarise(Mean = mean(rho), SD = sd(rho))
```

```
## # A tibble: 8 x 3
##   Measure   Mean    SD
##   <fct>    <dbl> <dbl>
## 1 Observed 0.455 0.301
## 2 Faiths   0.395 0.266
## 3 Shannon  0.573 0.274
## 4 BWPD     0.519 0.305
## 5 Jaccard  0.843 0.162
## 6 Unifrac  0.581 0.199
## 7 Morisita 0.650 0.312
## 8 Wunifrac 0.670 0.274
```

```r
results_i %>% group_by(Prevelance) %>%
  summarise(Mean = mean(rho), SD = sd(rho))
```

```
## # A tibble: 9 x 3
##   Prevelance  Mean    SD
##        <dbl> <dbl> <dbl>
## 1        0.1 0.837 0.159
## 2        0.2 0.758 0.203
## 3        0.3 0.706 0.229
## 4        0.4 0.656 0.249
## 5        0.5 0.604 0.262
## 6        0.6 0.557 0.271
## 7        0.7 0.464 0.291
## 8        0.8 0.353 0.286
## 9        0.9 0.336 0.239
```

```r
results_i %>%
  summarise(Mean = mean(rho), SD = sd(rho))
```

```
##        Mean       SD
## 1 0.5858397 0.294995
```
